# A functional interaction between GRP78 and Zika virus E protein

**DOI:** 10.1038/s41598-020-79803-z

**Published:** 2021-01-11

**Authors:** Sarawut Khongwichit, Wannapa Sornjai, Kunlakanya Jitobaom, Mingkwan Greenwood, Michael P. Greenwood, Atitaya Hitakarun, Nitwara Wikan, David Murphy, Duncan R. Smith

**Affiliations:** 1 Molecular Pathology Laboratory, Institute of Molecular Biosciences, Mahidol University, 25/25 Phuttamonthon 4 Road, Salaya, Nakhon Pathom, 73170 Thailand; 2 Bristol Medical School: Translational Health Sciences, Dorothy Hodgkin Building, University of Bristol, Bristol, BS1 3NY UK

**Keywords:** Cellular microbiology, Virus-host interactions

## Abstract

Zika virus (ZIKV) is a mosquito-transmitted virus that has caused significant public health concerns around the world, partly because of an association with microcephaly in babies born to mothers who were infected with ZIKV during pregnancy. As a recently emerging virus, little is known as to how the virus interacts with the host cell machinery. A yeast-2-hybrid screen for proteins capable of interacting with the ZIKV E protein domain III, the domain responsible for receptor binding, identified 21 proteins, one of which was the predominantly ER resident chaperone protein GRP78. The interaction of GRP78 and ZIKV E was confirmed by co-immunoprecipitation and reciprocal co-immunoprecipitation, and indirect immunofluorescence staining showed intracellular and extracellular co-localization between GRP78 and ZIKV E. Antibodies directed against the N-terminus of GRP78 were able to inhibit ZIKV entry to host cells, resulting in significant reductions in the levels of ZIKV infection and viral production. Consistently, these reductions were also observed after down-regulation of GRP78 by siRNA. These results indicate that GRP78 can play a role mediating ZIKV binding, internalization and replication in cells. GRP78 is a main regulator of the unfolded protein response (UPR), and the study showed that expression of GRP78 was up-regulated, and the UPR was activated. Increases in CHOP expression, and activation of caspases 7 and 9 were also shown in response to ZIKV infection. Overall these results indicate that the interaction between GRP78 and ZIKV E protein plays an important role in ZIKV infection and replication, and may be a potential therapeutic target.

## Introduction

Zika virus (ZIKV) is an enveloped virus classified in the genus *Flavivirus*, family *Flaviviridae*^1^ that is predominantly transmitted to humans by the bite of an infected *Aedes* species mosquito^2,3^, although other transmission routes including sexual transmission^4,5^ and mother to fetus transmission^6,7^ exist. Infection with ZIKV typically results in a mild febrile illness that may additionally be associated with manifestations including rash, conjunctivitis, myalgia and arthralgia^8,9^. The symptoms are generally self-limiting although more significant symptoms including neurological complications such as Guillain–Barré syndrome (GBS)^10^ and congenital Zika syndrome in newborns whose mothers were infected during pregnancy^6,7^ have been reported. ZIKV was first isolated in 1947 from a rhesus monkey in Zika Forest in Uganda^11^. In 2015, a large outbreak of ZIKV outbreak occurred in Brazil^8,12^ and ZIKV subsequently spread to many other countries in the Americas^13^. In 2016, the World Health Organization declared ZIKV a public health emergency of international concern due to the rapid emergence of ZIKV and the association with neurological conditions such as GBS and microcephaly^14^, although this has since ended.

Genetic analysis of different isolates of ZIKV shows that ZIKV can be classified into two main lineages, the African and Asian lineages^15^. The mature ZIKV virion consist of three structural proteins, the capsid, membrane and envelope proteins. The envelope (E) protein is a major component of the ZIKV virion surface, and plays an essential role in receptor binding during virus entry. ZIKV E protein contains three distinct domain, an N-terminal domain (domain I), an elongated finger-like structure (domain II) which is responsible for dimerization of E protein and also contains a hydrophobic fusion loop involved in membrane fusion, and immunoglobulin-like domain (domain III) which plays a key role in receptor binding during virus entry to host cell^16^. The first step in ZIKV infection process is the interaction between ZIKV and its receptor on host cell surface and several potential cell surface molecule have been identified as specific receptors for ZIKV such as dendritic cell-specific intercellular adhesion molecule-3-grabbing non-integrin (DC-SIGN) in dendritic cells and the AXL kinase receptor in skin fibroblasts, microglia and endothelial cells^17,18^. However, other studies have shown that AXL does not serve as ZIKV receptor in human astrocytes, but rather promotes ZIKV infection through suppression of type I IFN signaling^19^. Although the major role of envelope protein of ZIKV is receptor binding for virus entry to the host cell, ZIKV E protein might also have a functional role in manipulating host cellular pathways through interacting with host cell proteins to generate a favorable environment for viral replication^20^.

In the absence of a commercial vaccine to protect against ZIKV infection, an understanding of ZIKV interactions with host cell proteins is important for anti-viral drug development. In this study a yeast-2-hybrid screen of a human brain cDNA library was undertaken to identify ZIKV E protein interacting proteins. Interestingly, one of identified proteins from the yeast-2-hybrid screen, glucose regulated proteins 78 (GRP78), has been associated with replication of other flaviviruses, namely dengue virus (DENV^21–24^) and Japanese encephalitis virus (JEV^25,26^), as well as that of hepatitis A virus which belongs to the *Hepatovirus* genus of the *Picornaviridae* family^27,28^. The ER chaperone protein GRP78 is encoded by the HSPA5 gene and belongs to the heat shock protein 70 (HSP70) family^29^. However, GRP78 can be also expressed on the plasma cell surface^30^ and there have been reports that GRP78 can act as virus receptor for DENV^21^ and JEV^26^. GRP78 has an essential role to facilitating proper folding of nascent proteins, but moreover, GRP78 play a critical role in the cellular response to ER stress by mediating the unfolded protein response (UPR) pathway^31,32^. The UPR is cellular stress response which acts as a survival mechanism to restore ER homeostasis after ER stress by inhibiting protein loading to the ER, regulating the expression of ER chaperones and promoting increased protein degradation through the GRP78 mediated activation of three transmembrane signaling sensors namely, protein kinase RNA-like endoplasmic reticulum kinase (PERK), activating transcription factor 6 (ATF6) and Inositol-requiring kinase 1 (IRE1)^33^. Activation of the UPR in response to flavivirus infection has been reported for DENV^34,35^, JEV^36^, WNV^37^, and TBEV^38^. Under prolonged stress conditions in the ER, the UPR alters the pro-survival signal to a pro-cell death signal via the activation of CCAAT/-enhancer-binding protein homologous protein (CHOP) which is downstream gene target of PERK, leading to the induction of apoptosis^39,40^, a common consequence of flaviviruses infection^41–43^. Therefore, the second objective of this study was to investigate the activation of the UPR and apoptosis induction during ZIKV infection.

## Materials and methods

### ZIKV E protein yeast expression plasmid constructs

A plasmid harboring commercially synthesized DNA sequences encoded a partial ZIKV genome (Acc. No. KU955593, strain FSS13025, Cambodia isolated) was used as a template for ZIKV E gene amplification. This plasmid was commercially synthesized in 2015 as described elsewhere^44^. The specific primers used were (ZE forward) 5′-ATATGGCCATGGCAATCAGGTGCATAGGAGTCAGCAA-3′ and (ZE reverse) 5′-CCGCTGCAGCTAATCAGCAGAGACGGCTGTGGAT-3′. Cycle conditions were 98 °C for 10 s, and 30 cycles of 98 °C for 30 s, 30 s of 60 °C and 72 °C for 40 s and final extension at 72 °C for 5 min with Phusion DNA polymerase (Thermo Scientific Inc., Waltham, MA). The DNA fragments were ligated into the pGBKT7 yeast expression plasmid, generating the recombinant plasmid pGBKT7-ZE. The pGBKT7 plasmid contains a GAL4 DNA binding domain (GAL4-DNA-BD) and a c-myc fusion tag at the N-terminus, and also contains the TRP1 nutritional marker for selection in yeast.

Plasmid pGBKT7-ZE was subsequently used as a template to generate a ZIKV E protein domain III (ZEIII) construct using primers (ZEIII-*Nde*I-F) 5′-GGAATTCCATATGGATAAACTTAGATTGAAGGGCGTG-3′ and (ZEIII-*Pst*I-R) 5′-TTTTCTGCAGCTAACTCCTGTGCCAGTGGT-3′. DNA of ZEIII were amplified by Phusion^®^ High-Fidelity DNA polymerase (New England Biolabs, MA) using the same conditions as for amplification of full length ZIKV E. The PCR product was digested and ligated into the pGBKT7 yeast expression plasmid, generating the recombinant plasmid pGBKT7-ZEIII. The ligations were transformed to *E. coli* and the resultant clones were screened by restriction enzyme digestion. The clones that gave correct DNA fragment patterns were verified by commercial nucleotide sequencing.

### The cDNA library

The cDNA library used in this yeast-two hybrid assay was the Mate & Plate™ Library—Human Brain (Normalized) (Clontech Laboratories, Inc., CA). This cDNA library was constructed from human mRNA isolated from brain tissue of normal whole brains from 8 male Caucasians; ages between 43–66 years old with sudden death. The average cDNA size was 1.56 kb with size range of 0.7–3.0 kb. The titer was ≥ 5 × 10^7^ cfu/ml with 3.2 × 10^6^ independent clones.

### Yeast two-hybrid assay and screening

The Matchmaker^®^ Gold Yeast Two-Hybrid System (Clontech) was used to identify ZEIII protein interacting proteins. ZEIII (bait) was expressed as a fusion with the GAL4 DNA binding domain (GAL4-DNA-BD) in the Y2H Gold strain (reporter genes AUR1-C, HIS3, ADE2 and MEL1). While prey proteins (Human Brain Library) were expressed as a fusion to GAL4-AD in Y187 strain (reporter genes MEL1 and lacZ).

The yeast two-hybrid assay was performed according to the manufacturer’s protocol. Briefly, plasmid pGBKT7-ZEIII was transformed into Y2H Gold using a lithium acetate based method (Yeastmaker™ Yeast Transformation system 2, Clontech). The tryptophan positive clones were selected and concurrently assessed for autoactivation and toxicity. Subsequently, the level of ZEIII protein expression was investigated by Western blot analysis using an anti-c-myc tag antibody. To identify interacting proteins, a freshly prepared culture of the bait strain was mated with the Brain library. The cultures were screened by plating on medium lacking leucine and tryptophan, and containing X-α-Gal and Aureobasidin A (AbA). After an appropriate period of incubation, blue yeast colonies were patched on higher stringency selective medium (lacking adenine, leucine, histidine and tryptophan, and containing X-α-Gal and AbA) to confirm the positive interaction. To recover the plasmids from positive clones, yeast cells were resuspended in 50 µl of lyticase in TE buffer (5 U/µl, Invitrogen) followed by vigorous vortexing. Then the mixtures were incubated at 37 °C for 2 h with vortexing every 15 min. After that glass beads and 10 µl of 20% SDS were added into the mixtures followed by vigorous vortexing for 1 min. Then the extraction was continued using a plasmid extraction kit (Qiagen) according to the manufacturer’s protocol. Consequently, PCR amplification was performed using the recovered plasmids as the templates with the primers (T7 forward) 5′-TTAATACGACTCACTATAGGGC-3′ and (3′-AD reverse) 5′-CTGTGCATCGTGCACCATCT-3′ in the presence of 1 M betaine. The cycle conditions were 94 °C for 2 min, 35 cycles of 94 °C for 30 s, 60 °C for 30 s, 72 °C for 3 min and a final extension at 72 °C for 5 min with GoTaq^®^ DNA polymerase (Promega). The PCR products were electrophoretically separated on agarose gels to identify unique PCR products. After that, the plasmids were rescued by transformation into to *E. coli*, followed by plasmid extraction. These cDNA harboring plasmids were commercially sequenced by Eurofins Genomics Sequencing (Germany) and the genes were identified by nucleotide BLAST analysis. The resultant set of human proteins that were identified as interacting with ZIKV E protein were additionally submitted for analysis with using the STRING bioinformatics database for annotation and identification of known interactions between each protein.

### Cells and virus

A549 (human alveolar basal epithelial) cells were used throughout these experiments. The cells were grown and maintained in Dulbecco's modified eagle's medium (DMEM) (GIBCO, Invitrogen, Grand Island, NY), supplemented with 10% heat-inactivated fetal bovine serum (FBS) (Gibco, Invitrogen, USA), 1 × penicillin/streptomycin (PAA Laboratories GmbH, Pasching, Austria) at 37 °C with 5% CO_2_.

The ZIKV strains used in this study consist of strain SV0010/15 (ZIKV-T, Thai isolate) which was obtained from Armed Forces Research Institution of Medical Sciences (AFRIMS) and The Department of Disease Control, Ministry of Public Health, Thailand and ZIKV strain MR 766 (ZIKV-U) which was isolated from a nonhuman primate in Uganda in 1947. SV0010/15 was originally isolated from a Thai fever patient sample retrospectively screened and was passaged once in *Toxorhynchites splendens* mosquitoes followed by passage twice through C6/36 (*Aedes albopictus*) cells^45^. This virus was then passaged twice more through C6/36 cells, four times through LLC-MK2 cells^46^, and twice more through C6/36 cells before being used. Strain MR 766 is of uncertain passage history. Identity of both viruses was confirmed by partial DNA sequencing, and no differences from reported sequences were noted [GenBank accession numbers KX051562 (SV0010/15) and KY989511 (MR 766)].

### Plaque assay

Vero cells were seeded at 6 × 10^5^ cells/well in 6-well tissue culture plates, and then incubated under standard conditions until a 90% confluent monolayer was present within 24 h. The medium was removed and cells were subsequently inoculated with 200 µl of six different tenfold serially diluted (10^–2^–10^–7^) of stock virus or culture supernatant containing infectious viruses in BA-1 medium. The cells were then incubated at 37 °C for 2 h with gentle rocking every 10 min. After 2 h of viral absorption, cells were incubated with 4 ml of overlay medium, a mixture of 2 × nutrient solution and 0.8% (w/v) Seakem LE agarose in water (1:1) after which cells were maintained at 37 °C, 5% CO_2_ for 5 days. On the 6th day, the second overlay solution of 2 × nutrient solution containing 0.06% neutral red mixed with 0.6% Seakem LE agarose in water (1:1) was added to each well. The plaque number was counted and virus titer was calculated in plaque forming unit/ml (pfu/ml) on 7th day. All experiments were performed independently in triplicate, with duplicate plaque assay of each sample.

### ZIKV infection of A549 cells

A549 cells were cultured in 60 mm cell culture dishes (Corning) at a density that allowed approximately 80% confluency to be reached within 24 h. The cultured cells or siRNA transfected cells were either mock infected or infected with ZIKV-T or ZIKV-U in serum free medium at the selected multiplicity of infection (MOI) for 2 h with rocking every 10 min. After infection, the medium was removed and growth medium containing serum was added after which the cells were cultured under standard condition until required.

### Flow cytometry

At least 1 × 10^6^ cells of mock or ZIKV infected cells were harvested by centrifugation and were then blocked with 10% goat serum (Gibco BRL) with incubation on ice for 30 min, followed by washing twice with 1 ml of PBS and then cells were fixed with 4% paraformaldehyde in PBS for 20 min at room temperature. After washing twice with 1 ml of 1% BSA in PBS, mock or ZIKV infected cells were permeabilized with 0.2% Triton X-100 in 1% BSA for 10 min at room temperature after which the cells were washed twice with 1% BSA. Subsequently, the cells were incubated overnight with a 1:2 dilution of a pan-specific anti-flavivirus E protein monoclonal antibody, (HB112)^47^. After washing three times with 1% BSA, cells were incubated with a 1:40 dilution of a FITC conjugated goat anti-mouse IgG polyclonal antibody (KPL, Gaithersburg, MD) at room temperature for 1 h. After three washes with 1% BSA, cells were re-suspended in 400 μl of PBS and analyzed by flow cytometry (BD, FACSCalibur) using the CELLQuest™ software (BD Biosciences). All experiments were undertaken independently in triplicate.

### Co-immunoprecipitation (co-IP)

A549 cells were grown to 80% confluency in 100 mm^2^ tissue culture plates after which the cells were either mock infected or infected with ZIKV-T or ZIKV-U for 2 h. At 24 h post-infection (p.i.), cell lysates were prepared using IP lysis buffer [50 mM Tris–HCl pH 7.5, 150 mM NaCl, 1% NP-40, 0.5 mM EDTA, 0.5 mM activated Na_3_VO_4_ and 1 × protease inhibitor cocktail (PIC)]. Briefly, cells were washed twice with 1 × PBS before being resuspended in pre-cooled IP lysis buffer followed by mixing and centrifugation at 14,000*g* for 10 min at 4 °C. Next, the supernatant was transferred into a new tube and the protein concentration was measured by the Bradford assay. To immunoprecipitate ZIKV E protein or GRP78 from cell lysates, 1 mg of cell lysates were mixed with Protein G Sepharose 4 Fast Flow beads (GE Healthcare, Buckinghamshire, UK) and samples were rotated at 4 °C for 1 h to pre-clear the cell lysates. Subsequently, each sample of pre-cleared cell lysate was divided into two and transferred to new tubes. To immunoprecipitate the proteins, pre-cleared lysates from mock infection or ZIKV infection were incubated with 1 µg of a pan specific anti-flavivirus E protein mouse monoclonal antibody (HB112) or 1 µg of anti-GRP78 goat polyclonal antibody (sc-1050; Santa Cruz Biotechnology Inc., Dallas, TX) with gentle agitation overnight at 4 °C, while the second aliquot was incubated without antibody as a negative control. The mixtures were then incubated with 30 μl of protein sepharose bead slurry with gentle rocking for 4 h at 4 °C. After centrifugation at 6000×*g* for 3 min, the supernatant was discarded and pellets were then washed four times with IP lysis buffer. To elute the protein complexes, the pellet was resuspended in 30 μl of 3 × SDS sample buffer followed by heating at 100 °C for 5 min and then centrifugation at 14,000×*g* for 3 min at 4 °C. The supernatant was collected and analyzed by western blot analysis.

### Western blot analysis

Proteins were separated by electrophoresis through 12% or 15% sodium dodecyl sulfate–polyacrylamide gels and transferred to nitrocellulose membranes (Whatman GmbH, Germany). The membranes were subsequently blocked in blocking solution (5% (w/v) skimmed milk in Tris-buffered saline containing 0.05% Tween 20) for 30 min or 2 h at room temperature. The membranes were subsequently incubated overnight with shaking at 4 °C with a 1:1000 dilution of rabbit anti-GRP78 polyclonal antibody (sc-13968; Santa Cruz Biotechnology, Inc.), a 1:3000 dilution of a rabbit anti-Zika E protein polyclonal antibody (GTX133314; GeneTex, Inc., Irvine, CA), a 1:10,000 dilution of a rabbit anti-Zika NS1 protein polyclonal antibody (GTX133307; GeneTex, Inc.), a 1:1000 dilution of rabbit anti-caspase 9 polyclonal antibody (#9502, Cell Signaling Technologies, Danvers, MA), a 1:1000 dilution of a mouse anti-caspase 7 monoclonal antibody (9494, Cell Signaling Technologies), a 1:2500 dilution of a mouse anti-GAPDH monoclonal antibody (sc-32233, Santa Cruz Biotechnology, Inc.) or a 1:1000 dilution of a goat anti-actin (sc-1616; Santa Cruz Biotechnology, Inc). After washing, the membranes were incubated with an appropriate secondary antibody, namely a 1:5000 dilution of a HRP-conjugated mouse anti-rabbit IgG polyclonal antibody, a 1:6000 dilution of a HRP conjugated rabbit anti-goat IgG polyclonal antibody or a 1:10,000 dilution of a HRP-conjugated rabbit anti-mouse IgG polyclonal antibody at room temperature for 1–2 h. Finally, the signals were developed using the Luminata TM Forte Western HRP Substrate (Merck Millipore, Burlington, MA) and signal was detected by using autoradiography film or an Azure c400 Gel Imaging System (Azure Biosystems chemiluminescence, Azure Biosystems, Inc., Dublin, CA).

### RNA extraction, semi-quantitative and quantitative RT-PCR analysis

Total RNA of cells or the supernatant was extracted using Trizol reagent (Molecular Research Center, Cincinnati, OH) according to the manufacturer’s instructions. The extracted RNA from cells was treated with DNaseI (RNase-free) (Ambion) while extracted RNA from culture supernatant was directly used as the template for cDNA synthesis without DNaseI treatment. The cDNA was reverse transcribed from 1 µg of total cellular RNA or 5 µl of extracted RNA from supernatant using RevertAid Reverse Transcriptase (Thermo Fisher Scientific Inc.,) and oligo dT (Bio Basic, Inc., Markham, Canada) or random hexamers (Thermo Fisher Scientific, Inc.).

Next, cDNA was subjected to semi-quantitative PCR for XBP1 and real time PCR for determination of the expression levels of GRP78, CHOP and actin using specific primers and PCR reaction conditions as described elsewhere^34,48^.

For quantitative PCR of ZIKV genome copy number, a DNA standard was prepared using conventional PCR amplification of the ZIKV envelope gene. The PCR reaction was comprised of cDNA derived from ZIKV strain SV0010/15, 1 × DreamTaq buffer, 0.25 µM of each primer; ZIKE_RT_Fw2 5′-TTGGAGGAATGTCCTGGTTCTCAC-3′ and ZIKE_RT_RV2 5′-AGTCAGGATGGTACTTGTACC-3′, 200 µM dNTPs and 0.025 U/µl of DreamTaq DNA polymerase (Thermo Fisher Scientific). The amplification was carried out under the following conditions; 95 °C for 3 min and followed with 30 cycles of denaturation at 95 °C for 10 s, annealing at 60 °C for 30 s, extension at 72 °C for 20 s and the final extension at 72 °C for 5 min. The PCR product was purified using a FavorPrep PCR purification mini kit (FAVORGEN Biotech Corporation, Ping-Tung, Taiwan) following the manufacturer’s protocol. The PCR product concentration was measured by using a Nanodrop 2000 and the DNA copy number was calculated using the online Thermo Fisher Scientific web calculator. After that, a tenfold serial dilutions were prepared to obtain 10^2^–10^10^ copies DNA as a standard control. The ZIKV genome copy number was quantitated using real time PCR and the reaction was comprised of 2.5 µl of either diluted cDNA sample or standard controls, 1 × KAPA SYBR FAST Master Mix Universal, 0.3 µM of ZIKE_RT_Fw2 and ZIKE_RT_RV2 primer. The amplification was undertaken using a Mastercycler Realplex (Eppendorf, Hauppauge, NY). The standard curve was generated from Ct cycle of the DNA standard controls and the ZIKV genome copy number was calculated using the Mastercycler Realplex software.

### Immunofluorescence assay

A549 cells were grown on glass cover slips at a density that allowed 80% confluency to be reached within 24 h, after which the cells were either mock infected or infected with ZIKV-T and ZIKV-U at MOI 5 and MOI 2, respectively for 1 h at 4 °C. The cells were then washed twice with PBS and fixed with 1% paraformaldehyde in 1 × PBS for 20 min. After removal of the fixing reagent, cells were then blocked with 5% BSA in 0.03% Triton X-100 in PBS for 20 min follow by incubation with two different primary antibodies at 4 °C overnight. After washing four times, the cells were incubated with two appropriate secondary antibodies at room temperature for 1 h. Subsequently, cells were washed five times with 0.03% Triton X-100 in PBS and mounted onto glass cover slips using Prolong^®^ Gold antifade reagent (Invitrogen, Thermo Fisher Scientific Inc.).

For intracellular co-localization analysis, A549 cells were seeded on glass cover slips and cultured under standard conditions for 1 day. The cells were subsequently mock infected and infected with ZIKV-T or ZIKV-U. On 1 day p.i., cells were fixed with 4% paraformaldehyde in 1 × PBS for 20 min. After washing, cells were then permeabilized with 0.3% Triton X-100 in PBS for 10 min before blocking with 5% BSA in 0.03% Triton X-100 for 20 min. Cells were subsequently incubated with one or two appropriate primary antibodies at 4 °C overnight after which cells were washed four times with 0.03% Triton X-100 in PBS follow by incubating with one or two appropriate secondary antibodies and DAPI for nuclear staining at room temperature for 1 h. Next, cells were washed five times to remove unbound or excess antibody followed by mounting with Prolong^®^ Gold antifade reagent (Invitrogen, Thermo Fisher Scientific Inc.) onto glass slides before visualization under a confocal microscope.

Primary antibodies used included a 1:200 dilution of rabbit anti-Zika E protein polyclonal antibody (GTX133314; GeneTex, Inc.), a 1:2 dilution of a mouse monoclonal pan-specific anti-flavivirus E protein antibody (HB112), a 1:20 dilution of a goat polyclonal anti-GRP78 antibody (sc-1050; Santa Cruz Biotechnology, Inc). Secondary antibodies used were a 1:50 dilution of a FITC-conjugated donkey anti-rabbit IgG antibody (sc-2090; Santa Cruz Biotechnology, Inc.), a 1:200 dilution of an Alexa 488-conjugated donkey anti-mouse IgG antibody (A21202; Molecular Probes, Thermo Fisher Scientific Inc.), a 1:50 dilution of an Alexa 568-conjugated donkey anti-goat IgG antibody (A11057; Molecular Probes, Thermo Fisher Scientific Inc.), and a 1:100 dilution of a rhodamine red X-conjugated goat anti-rabbit IgG antibody (Jackson ImmunoResearch Laboratories). The fluorescent signals were observed under a ZEISS LSM 800 with Airyscan confocal microscope (Carl Zeiss, Jena, Germany) using the ZEN software. The degree of co-localization was determined as Pearson correlation coefficients using ImageJ software with JACoP plugin as described elsewhere^49^. Pearson correlation coefficients were undertaken for at least 20 cells from representative fields for each condition.

### Antibody mediated infection inhibition assay

A549 cells were seeded into 6-well plate and grown under standard condition until the cells reached 80% confluence within 24 h. Next, the cells were incubated with or without various concentrations of a rabbit polyclonal anti-GRP78 N-terminus antibody (ab32618; Abcam) or a rabbit polyclonal anti-GRP78 C-terminus antibody (ab21685; Abcam) or 20 μg of a rabbit polyclonal anti-p-PERK (Thr981) antibody (sc-32577-R; Santa Cruz Biotechnology Inc.) at 37 °C for 1 h. After incubation, the cells were washed with 1 × PBS and cells were then infected with ZIKV-T at MOI 5 at 37 °C for 2 h following which excess virus was removed by washing three times with 1 × PBS and fresh growth media was added. At 18 h p.i., cells were harvested and ZIKV infection was determined by flow cytometry, and the viral titer in the supernatant was analyzed by standard plaque assay as described above. All experiments were undertaken independently in triplicate.

### siRNA mediated gene silencing of GRP78

GFP siRNA and GRP78 siRNA were constructed using oligonucleotide templates; GFP siRNA-F 5′-AACTTGTAGTTCCCGTCATCTCCTGTCTC-3′, GFP siRNA-R 5′-AAAGATGACGGGAACTACAAGCCTGTCTC-3′, GRP78 siRNA-F 5′-AACATTTATTGGTGTCACTTATGGCCTGTCTC-3′ GRP78 siRNA-R 5′-CCATAAGTGACACCAATAAATGTTCCTGTCTC-3′ and the Silencer siRNA Construction Kit following the manufacturer’s instructions (Life Technologies, Carlsbad, CA). A549 cells were grown in 12-well plate at a density to reach 60% confluence within 24 h. DMEM containing 10% FBS was removed and the cells were either transfected with 60 pM GFP siRNA or GRP78 siRNA using 1 µl of Lipofectamine RNAiMAX (Life Technologies, Carlsbad, CA). Transfected cells were incubated under standard conditions at 37 °C with 5% CO_2_ for 24 h.

### ZIKV binding and/or entry assay

After 24 h of GRP78 or GFP knockdown by siRNA, transfected A549 cells media was replaced with 1 ml of cold FBS free DMEM and cells were incubated on ice for 10 min. For viral binding assay, cells were incubated with ZIKV-T at m.o.i. of 5 or 200 at 4 °C for 1 h. For viral binding and entry assay, A549 cells were incubated with ZIKV-T at m.o.i. 5 at 37 °C with 5% CO_2_ for 2 h. After incubation, the unbound virus was removed from cells by washing with ice cold 1 × PBS three times. Washed cells were collected for RNA extraction and determination of ZIKV genome copy number.

### Statistical analysis

All data were plotted using the GraphPad Prism 5 program (GraphPad Software, Inc., CA). Evaluation of protein co-localization was undertaken by determining Pearson's correlation coefficients exactly as described elsewhere^49^. The statistical analysis was undertaken by independent *t* test using SPSS (SPSS Inc.) with a *p*-value ≤ 0.05 taken as significant.

## Results

### Identification of ZIKV E protein interacting proteins using yeast two-hybrid system

**Figure 1:**
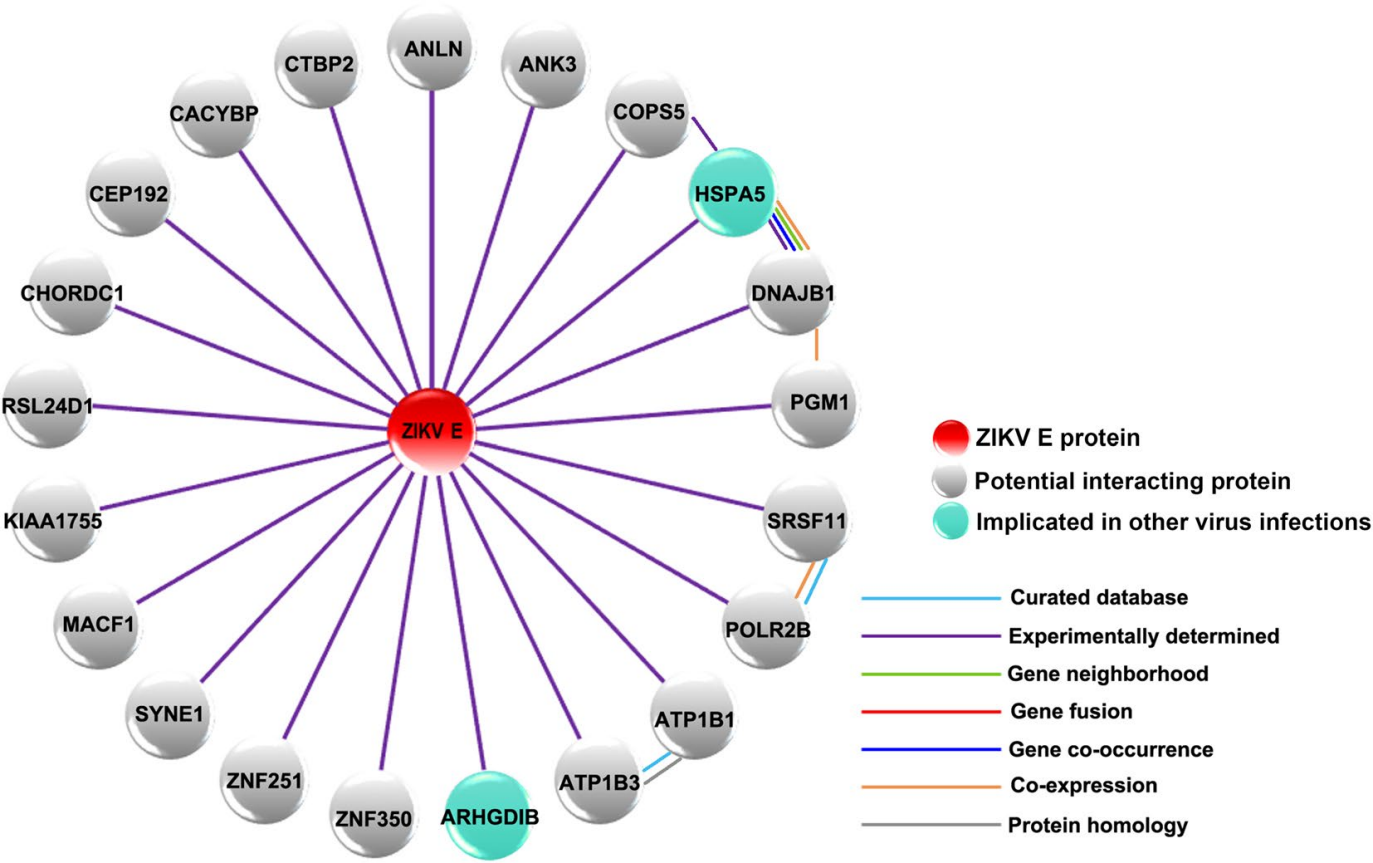
The interaction of ZIKV E protein and human proteins. This diagram represents the set of potential ZIKV E protein domain III interacting proteins that were identified by a yeast two-hybrid experiment. The red circle indicates ZIKV E protein, grey circles indicate human proteins and light green circles indicate interacting proteins that have been previously implicated in replication of other viruses. For the interaction between each protein, light blue lines indicate interactions with evidence from curated databases, and purple lines indicate that the interaction was experimentally determined. The predicted interactions are indicated, green lines represent gene neighborhood, red lines represent gene fusion, and blue lines represent gene co-occurrence. Orange lines indicate co-expression and grey lines indicate protein homology.

To identify ZIKV E protein interacting proteins, we initially attempted to use a full length ZIKV bait protein. However, the full length protein was highly toxic to the yeast cells, resulting in low efficiency of mating of the bait and prey libraries (Supplemental Table S1). To reduce the toxicity to yeast, domain III of ZIKV E protein was subcloned and used in subsequent yeast-2-hybrid experiments. Therefore the Y2H gold yeast strain expressing domain III of ZIKV E protein was mated with Y187 strain containing a human brain cDNA library. The first screening yielded 200 positive blue colonies. The blue colonies were patched on higher stringency media to confirm the positive clones, and only 2 colonies did not grow. The remaining 198 positive colonies which all grew on quadruple knock out plates [SD/-Ade/-His/-Lue/-Trp/X-α-gal/AbA (QDO/X/A)] were subjected to plasmid extraction and further screened by PCR to remove duplicates. Subsequently, a number of plasmids were transformed into *E. coli* to rescue the plasmids, which were then subjected to DNA sequencing. From 198 positive colonies, 87 plasmid samples were sequenced, and 35 unique potential interacting proteins were identified (some interacting candidates were identified multiple times). Of the 35 interacting candidates, 14 false positives were obtained as the DNA sequences matched to the untranslated region (UTR) of genes (data not shown). Thus, 21 potential interacting proteins were identified in this study (Table 1). The set of human proteins that were identified to interact with ZIKV E protein were submitted for analysis using the STRING bioinformatics database^50^. The STRING analysis identified the ZIKV E protein interacting proteins as being involved in several biological processes including regulation of ATPase activity, cellular sodium–potassium ions homeostasis and maintenance of protein location in the cell and membrane repolarization (Supplemental Table S2). However, the overall protein–protein interaction (PPI) enrichment p-value was low (PPI enrichment p-value = 0.262), suggesting little functional connection between the identified proteins. The ZIKV E protein-human protein interactome is shown in Fig. 1. Of the 21 proteins identified, GRP78 was of particular interest as it has been shown to directly interact with the E protein of both DENV^21–24^ and Japanese encephalitis virus^25,26^. This interaction was therefore selected for validation and further investigation.

**Table 1:** Identified ZIKV E protein domain III interacting proteins from yeast two-hybrid assay.

Gene name	Descriptions	Accession no
ANLN	Anillin actin binding protein	NM_001284302.2
ANK3	Ankyrin 3	NM_020987.4
ARHGDIB	Rho GDP dissociation inhibitor beta	NM_001175.6
ATP1B1	ATPase Na^+^/K^+^ transporting subunit beta 1	NM_001677.3
ATP1B3	ATPase Na^+^/K^+^ transporting subunit beta 3	NM_001679.3
CACYBP	Calcyclin binding protein	NM_014412.2
CEP192	Centrosomal protein 192	NM_032142.3
CHORDC1	Cysteine and histidine rich domain containing 1	NM_001144073.1
COPS5	COP9 signalosome subunit 5	NM_006837.2
CTBP2	C-terminal binding protein 2	NM_001329.3
DNAJB1	DnaJ heat shock protein family (Hsp40) member B1	NM_001313964.1
HSPA5	Heat shock protein family A (Hsp70) member 5, GRP78	NM_005347.4
KIAA1755	KIAA1755	NM_001029864.1
MACF1	Microtubule-actin crosslinking factor 1	XM_005270694.1
PGM1	Phosphoglucomutase 1	NM_002633.2
POLR2B	RNA polymerase II subunit B	NM_001303268.1
RSL24D1	Ribosomal L24 domain containing protein 1	NM_016304.2
SRSF11	Serine and arginine rich splicing factor 11	NM_004768.3
SYNE1	Spectrin repeat containing nuclear envelope protein 1	NM_182961.3
ZNF251	Zinc finger protein 251	NM_138367.1
ZNF350	Zinc finger protein 350	NM_021632.3

### ZIKV infection of A549 cells and viral production^46,51^

**Figure 2:**
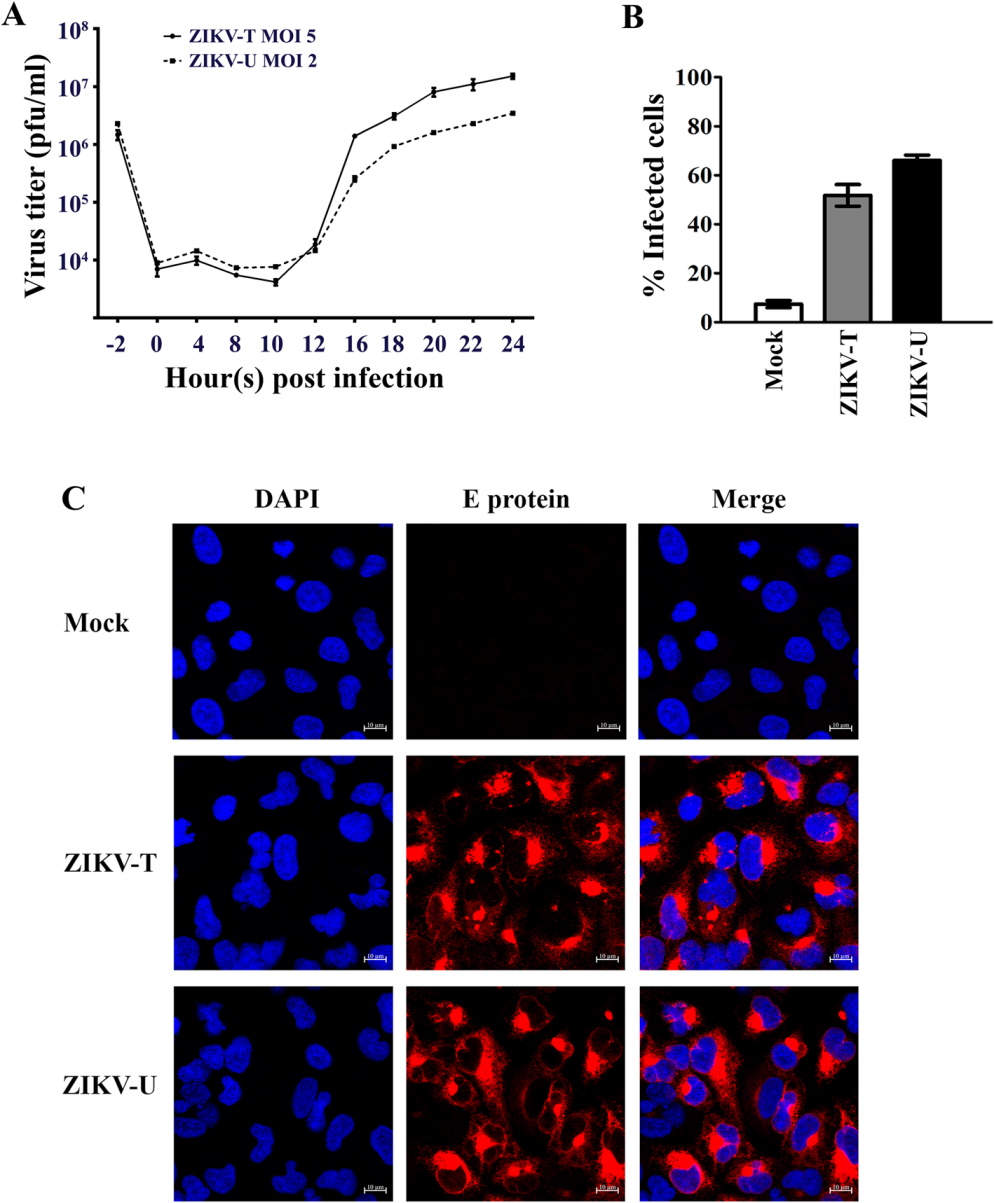
Zika virus infection of A549 cells**.** A549 cells were mock-infected or infected with ZIKV-T or ZIKV-U at MOI 5 or 2, respectively before determining (**A**) the viral titer by plaque assay on Vero cells at the indicated time points post infection (p.i), with the plot showing the results of independent triplicate experiments, with duplicate plaque assay of samples, and (**B**) the infection levels by flow cytometry on 1 d p.i., with the plot represent means ± SEM of three independent replicates, or (**C**) expression of ZIKV E protein at 24 h.p.i was determined by confocal microscopy after staining using an anti-ZIKV E protein rabbit polyclonal antibody followed by a rhodamine red X-conjugated goat anti-rabbit IgG antibody (red) and DAPI (blue) for nuclei staining. Fluorescent signals were observed under a confocal microscope (ZEISS).

Before validating the interaction between GRP78 and ZIKV E, the profile of viral productivity and infectivity for two isolates of ZIKV after infection of A549 cells was determined. Based on our previous study which shows different levels of infectivity resulting from similar MOIs for ZIKV-T and ZIKV-U^46^, A549 cells were mock infected or infected with ZIKV-T at MOI 5 and ZIKV-U at MOI 2. A549 cells were selected as our previous studies have shown that this cell line has good susceptibility to ZIKV^46,51^. At the indicated time points, the culture supernatant and cells were harvested to determine virus titer and level of infection, respectively. Plaque assay was used to measure the viral titer of ZIKV-T and ZIKV-U in the culture supernatant at -2 (addition of virus to cells), 0, 4, 8, 10, 12, 14, 16, 18, 20, 22, 24 h post infection. The results (Fig. 2A) showed the virus titer decreasing over the first 12 h p.i. consistent with virus adsorption and/or entry followed by de novo virus production starting at around 12–16 h.p.i, with highest titer at 24 h p.i. At 24 h p.i. flow cytometry analysis showed (Fig. 2B) that both ZIKV-T and ZIKV-U infection resulted in 50–60% of cells being infected. Infection with the African isolate resulted in slightly more cells being infected, even though cells were infected using a lower MOI (ZIKV-T MOI 5, ZIKV-U MOI 2). Visualization of infection by immunofluorescence at 24 h.p.i using the same MOIs (ZIKV-T MOI 5, ZIKV-U MOI 2) confirmed robust levels of infection (Fig. 2C), which appeared somewhat higher than the level observed by flow cytometry.

### Expression of GRP78 during ZIKV infection

**Figure 3:**
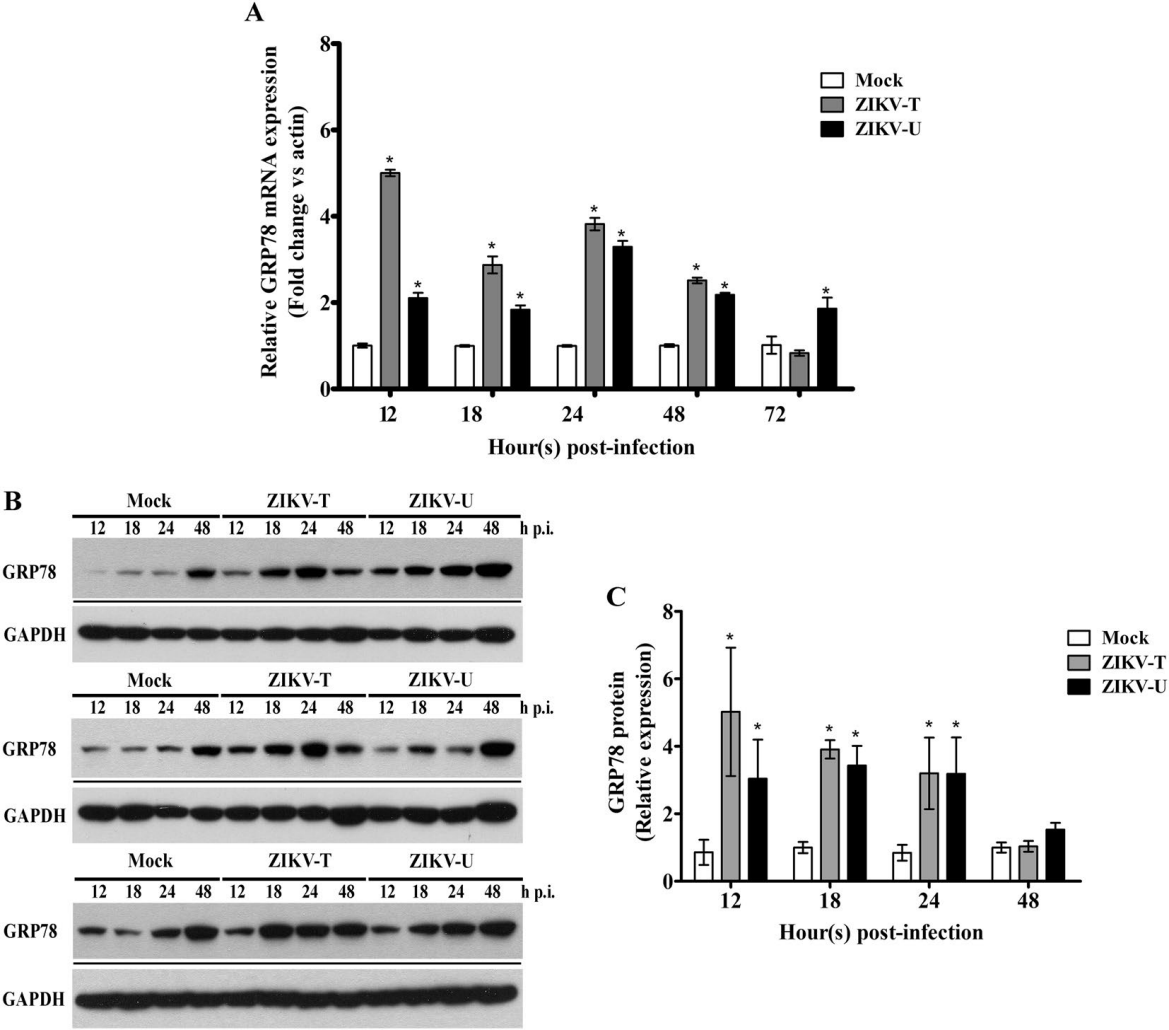
The expression of GRP78 during ZIKV infection. A549 cells were mock infected or infected with ZIKV-T and ZIKV-U at MOI 5 or MOI 2 respectively, and analyzed for (**A**) levels of expression of GRP78 mRNA by quantitative real time PCR. The relative expression levels of GRP78 mRNA were normalized against actin using the comparative CT method (2^−ΔΔCT^ method), or (**B**) levels of expression of GRP78 protein by western blot analysis with GAPDH expression as an internal loading control followed by (**C**) determination of band intensities by Quantity One software. All experiments were performed independently in triplicate. Error bars show SEM (*p value < 0.05). For (**B**) repeat probings of the same filter are shown separated by a black line, and uncropped blots are presented in Supplemental materials.

To investigate the expression of GRP78 in response to ZIKV infection, A549 cells were either mock infected or infected with ZIKV-T at MOI 5 or ZIKV-U at MOI 2. At various time points, cells were harvested, and subjected to RNA or protein extraction as appropriate. The expression of GRP78 mRNA and protein were determined using quantitative real time RT-PCR and western blot analysis, respectively. Quantitative real time PCR analysis (Fig. 3A) showed that GRP78 mRNA expression levels were significantly increased by ZIKV infection with both strains as compared to the mock infected control. Similarly, western blot analysis showed increased protein expression as compared to mock for both ZIKV strains examined from 12 h p.i. to 24 h p.i. (Fig. 3B,C).

### The interaction of GRP78 and ZIKV E protein

**Figure 4:**
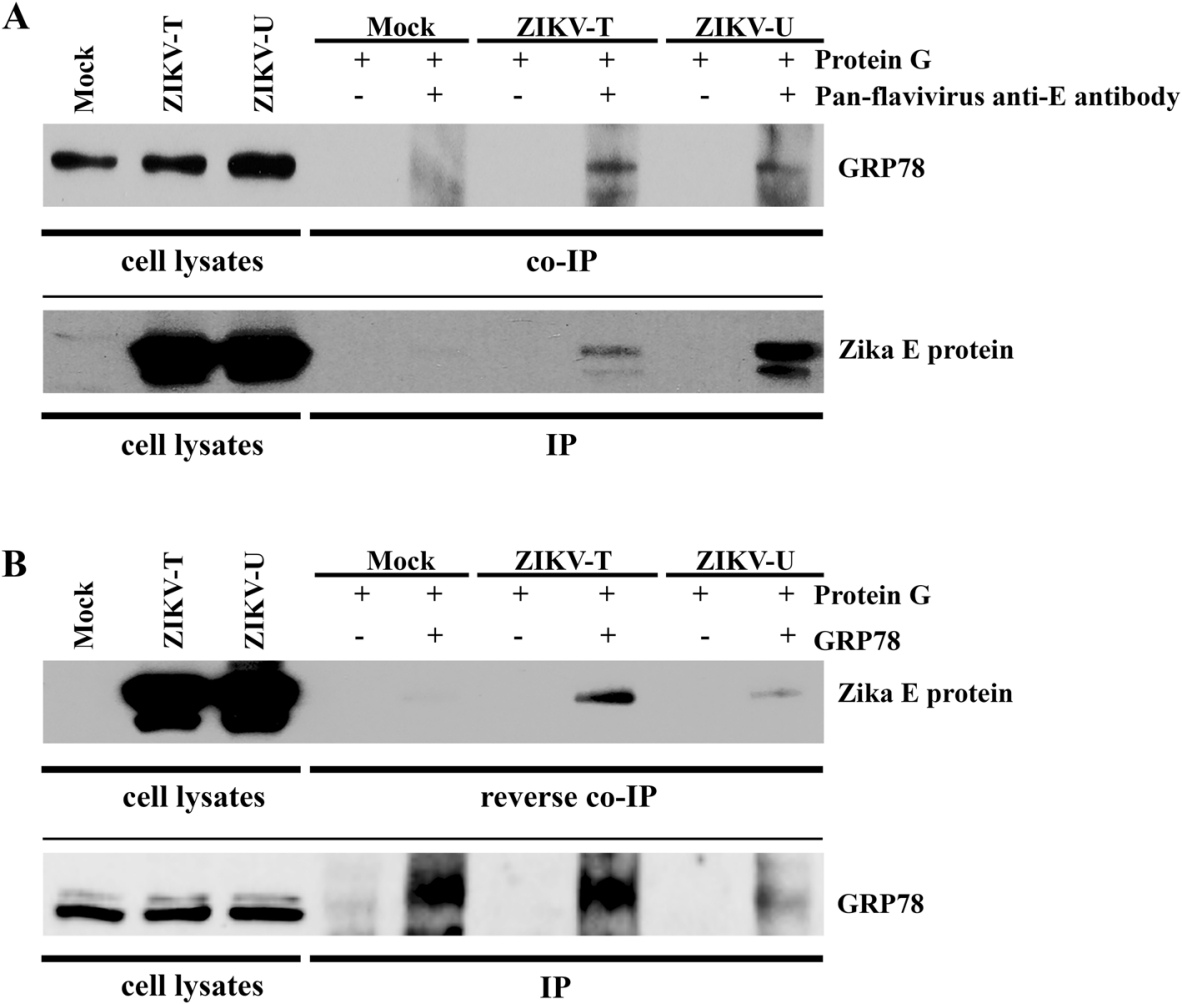
Investigation of the interaction between GRP78 and ZIKV E protein. A549 cells were mock-infected or infected with ZIKV-T or ZIKV-U at MOI 5 or 2, respectively and cell lysates were collected on day 1 post infection. (**A**) Immunoprecipitation was performed using a pan specific anti-flavivirus E protein antibody (HB112) for pull down followed by western blot analysis with an anti-GRP78 antibody. To confirm the immunoprecipitation of ZIKV E protein, the membrane was re-probed with anti-ZIKV E antibody. (**B**) Reverse co-immunoprecipitation was performed using an anti-GRP78 antibody for pull down followed by western blot analysis with an anti-flavivirus E protein antibody. The membrane then re-probed with an anti-GRP78 antibody to confirm the immunoprecipitation of GRP78 protein. For (**A**) and (**B**) repeat probings are separated by a continuous black line and uncropped blots are presented in Supplemental materials.

To investigate the interaction of GRP78 and ZIKV E protein, a co-immunoprecipitation assay was performed. A549 cells were mock infected or infected with ZIKV-T or ZIKV-U at MOI 5 and MOI 2, respectively. On 1 day post infection, cells were harvested for protein extraction and the resultant lysates were incubated with or without a pan specific anti-flavivirus E protein antibody (HB112). The precipitated protein complexes were separated by electrophoresis before transfer to nitrocellulose membranes. Subsequently, the membrane was probed with a rabbit polyclonal anti-GRP78 antibody to detect the co-immunoprecipitation of GRP78. The results (Fig. 4A) show that GRP78 was co-immunoprecipitated together with ZIKV E protein from both strains investigated (ZIKV-T and ZIKV-U). Reprobing of the membrane with an antibody directed against ZIKV E protein confirmed the pull down of ZIKV E protein.

To further confirm the interaction between GRP78 and ZIKV E protein, a reverse co-immunoprecipitation was performed. This time GRP78 was pulled down from infected and mock infected lysates and the membrane was probed with an antibody directed against ZIKV E protein, and again the membrane was subsequently probed with an antibody directed against the pull down protein (GRP78). Results (Fig. 4B) show that the E proteins of ZIKV-T and ZIKV-U were co-immunoprecipitated with GRP78, confirming the interaction of Zika E protein and GRP78.

### Co-localization of GRP78 and ZIKV E protein

**Figure 5:**
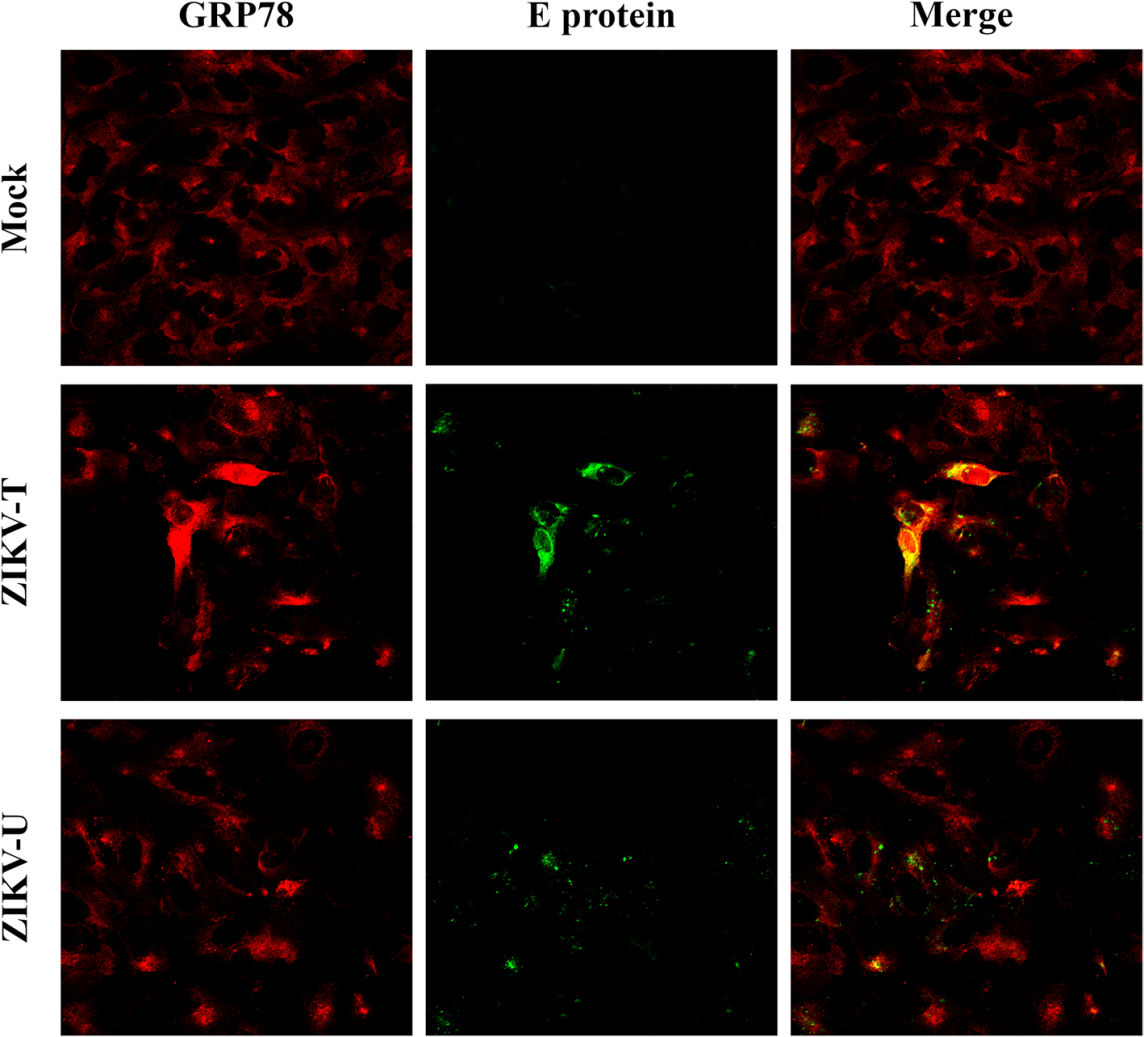
Colocalization analysis of GRP78 and ZIKV E protein on the cell surface. A549 cells were grown on glass slides and then incubated with ZIKV-T or ZIKV-U at 4 °C for 1 h. Under non-permeabilization conditions, cells were strained with primary antibodies followed by appropriate secondary antibodies. The cell surface colocalization (yellow) between GRP78 (red) and ZIKV E protein (green) was determined using a ZEISS confocal microscope.

To determine whether there is cell surface co-localization between GRP78 and ZIKV E, A549 cells were incubated with ZIKV-T and ZIKV-U for 1 h at 4 °C and were then fixed with 1% paraformaldehyde. A no virus control was undertaken in parallel. The non-permeabilized cells were incubated with a mouse monoclonal pan-specific anti-flavivirus E protein and a goat polyclonal anti GRP78 antibody. Results, Fig. 5, showed abundant GRP78 expression on the cell surface and co-localization between GRP78 and ZIKV E was observed in both ZIKV-T (mean Pearson correlation coefficient 0.54, P < 0.001) and ZIKV-U (mean Pearson correlation coefficient 0.42, P < 0.001) infected cells.

**Figure 6:**
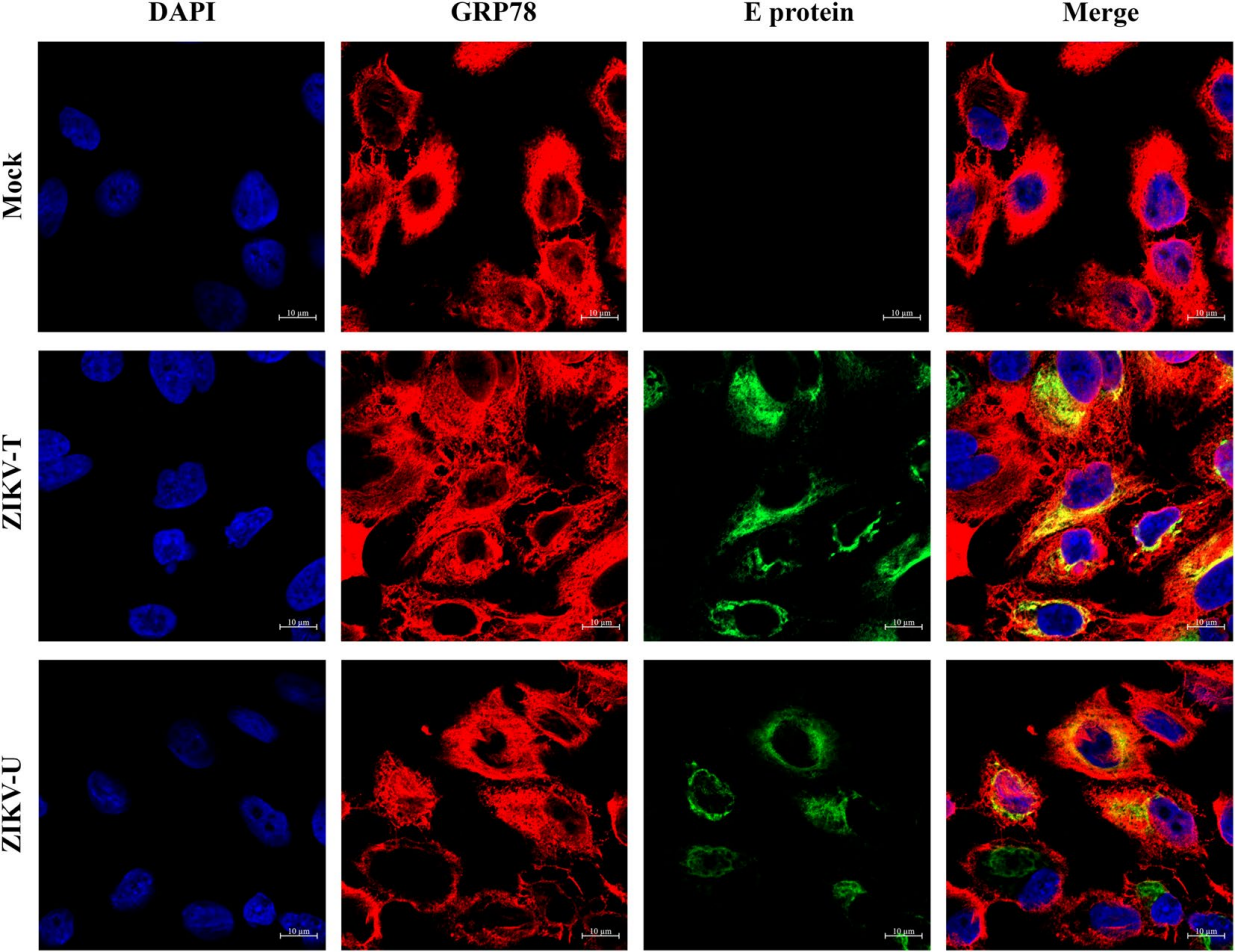
Intracellular colocalization of GRP78 and ZIKV E protein. A549 cells grown on glass slides were mock infected or infected with ZIKV-T and ZIKV-U. At 24 h p.i., cells were permeabalized and stained with an anti-GRP78 antibody and a pan specific anti-flavivirus E antibody follow by staining with an appropriate secondary antibodies and DAPI (blue) for nuclei visualization. Localization (yellow) between GRP78 (red) and ZIKV E protein (green) was observed under a ZEISS confocal microscope.

For analysis of the intracellular colocalization between GRP78 and ZIKV E protein, A549 cells were mock infected or infected with ZIKV-T or ZIKV-U and at 24 h post infection cells were fixed, permeabilized and incubated with primary antibodies directed against ZIKV E protein and GRP78. Results, Fig. 6, showed high levels of expression of GRP78, and co-localization between GRP78 and ZIKV E protein of both strains (ZIKV-T: mean Pearson correlation coefficient 0.495, P < 0.001; ZIKV-U: mean Pearson correlation coefficient 0.35, P < 0.001).

### Inhibition of ZIKV infection by antibody blocking cellular surface GRP78

**Figure 7:**
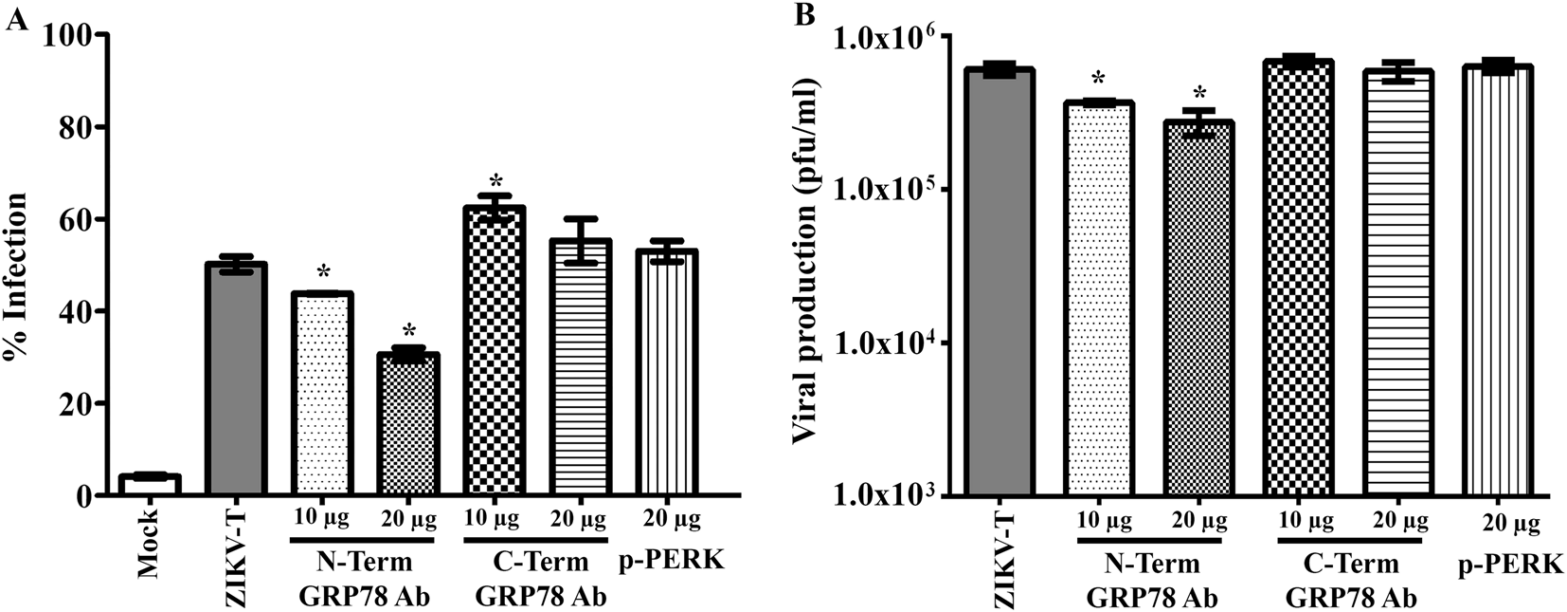
Antibody mediated inhibition of ZIKV-T infection of A549 cells. A549 cells were pre-treated with no antibody as a control or with anti-GRP78 N- and C-terminus antibodies at varying concentrations, or with 20 µg of a rabbit polyclonal anti-phospho-PERK antibody as a non-relevant antibody followed by ZIKV-T infection at MOI 5. At 18 h p.i., (**A**) the cells were collect to determine the percentage of cell infection by flow cytometry and (**B**) the culture supernatant was collected to determine viral titer by standard plaque assay. The graphs are representative of three independent biological replicates with duplicate plaque assay. Error bars represent SEM. (*p value < 0.05).

To determine whether GRP78 plays a role in mediating the internalization of ZIKV to A549 cells, A549 cells were pre-incubated with varying amounts of a rabbit polyclonal antibody directed to the N- or C-terminus of GRP78, or with a rabbit polyclonal antibody directed to p-PERK as a non-relevant control antibody, and cells were then infected with ZIKV-T. At 18 h p.i, cells and culture supernatants were harvested to determine the percentage ZIKV infection by flow cytometry, and the virus titer by standard plaque assay, respectively. Results showed that pre-incubation with an antibody directed against the N-terminus of GRP78 reduced the number of infected cells (Fig. 7A) as well as the virus titer (Fig. 7B). An increase of the level of infection (Fig. 7A) was seen in cells pre-incubated with 10 μg an antibody directed against the C-terminus of GRP78, but this was not seen with incubation with a higher concentration of this antibody (20 μg) and there was no significant difference observed in the virus titer (Fig. 7B). Similarly, pre-incubation with a non-relevant antibody had no effect upon either the degree of infection (Fig. 7A) or the virus titer (Fig. 7B).

### Effect of downregulation of GRP78 on ZIKV infection

**Figure 8:**
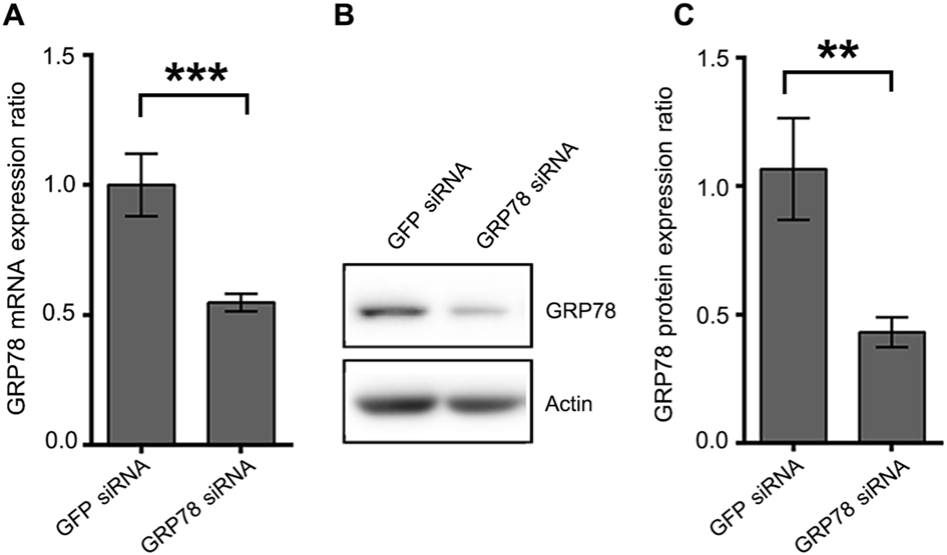
GRP78 knockdown of A549 cells. A549 cells were either transfected with GFP siRNA or GRP78 siRNA for 24 h. (**A**) The expression of GRP78 mRNA was quantitated by real-time PCR. The relative expression levels of GRP78 mRNA were normalized against GFP siRNA transfection control using the comparative CT method (2^−ΔΔCT^ method). (**B**) The expression of GRP78 protein was determined by western blot analysis. (**C**) The band intensity of detected proteins were quantitated by Quantity One software. The expression level of GRP78 protein was normalized against GFP siRNA transfection control and shown as the relative expression ratio. The graphs are representative of three independent biological replicates. Error bars represent SD (**p value < 0.01 and ***p value < 0.001).

The functional role of both cellular and intracellular GRP78 in ZIKV infection and replication was examined using siRNA mediated gene silencing. A549 cells were either transfected with siRNA directed to GRP78 or with a non-relevant control siRNA (GFP siRNA). After 24 h of transfection, GRP78 mRNA expression level was determined by quantitative real time PCR. The results showed that GRP78 mRNA was significantly reduced by approximately 45% by GRP78 siRNA transfection when compared to the GFP siRNA transfection control (Fig. 8A). In addition, the expression level of GRP78 protein was examined and the results showed an approximately 55% reduction of GRP78 expression in GRP78 siRNA transfected cells when compared to the GFP siRNA transfection control (Fig. 8B,C). These results indicated that GRP78 gene was successfully down regulated by siRNA mediated gene silencing.

**Figure 9:**
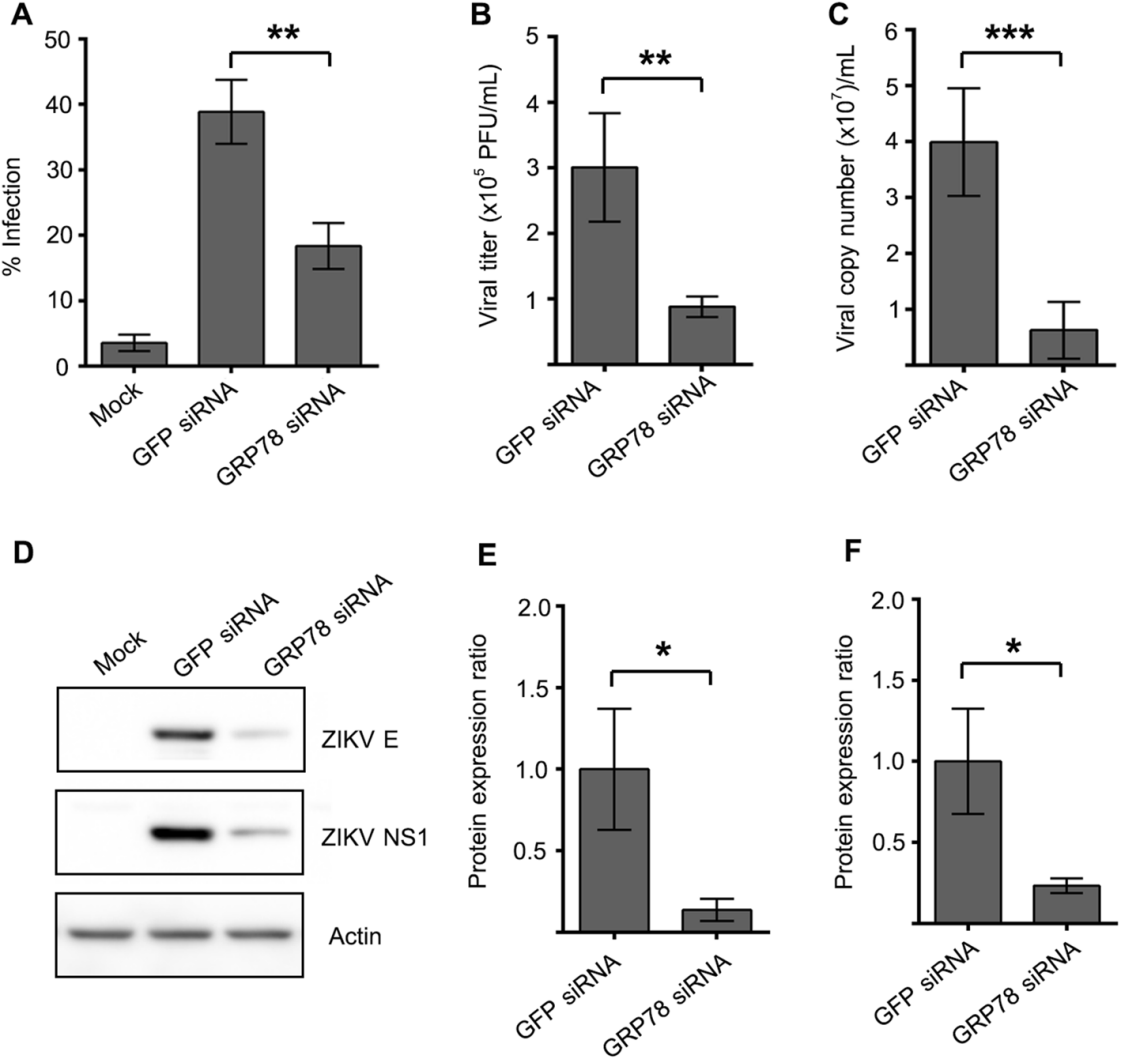
Effect of GRP78 knockdown to ZIKV infection and production. A549 cells were either transfected with GFP siRNA or GRP78 siRNA for 24 h. (**A**) The transfected cells were infected with ZIKV-T at m.o.i. of 5 for 24 h. (**A**) The infection level was determined by flow cytometry. The supernatant was collected from infected cells. (**B**) ZIKV titer and (**C**) ZIKV genome copy number were quantitated by standard plaque assay and real-time PCR, respectively. (**D**) ZIKV E and NS1 protein was detected by western blot analysis. The band intensity of (**E**) ZIKV E and (**F**) NS1 protein was quantitated by Quantity One software and then normalized against GFP siRNA transfection control. The protein expression level is presented as the relative expression ratio. The graphs are representative of three independent biological replicates. Error bars represent SD (*p value < 0.05, **p value < 0.01 and ***p value < 0.001).

To determine the effect of GRP78 knockdown to ZIKV infection, A549 cells were either transfected with GRP78 siRNA or GFP siRNA for 24 h and then subsequently infected with ZIKV-T at m.o.i. 5 for 24 h, after which the infection level was determined by flow cytometry analysis. From result, ZIKV infection in GRP78 siRNA transfected cells was significantly decreased by 47% when compared to infection of the GFP siRNA transfection control (Fig. 9A). At 24 h of infection, the culture supernatant was collected from infected cells and viral production was determined by plaque assay, while genome copy number was determined by and quantitative real time PCR. The results showed a significant reduction of viral titer and viral genome copy number after GRP78 knockdown as compared to the GFP siRNA transfection control (Fig. 9B,C). Furthermore, the expression of ZIKV proteins in the infected cells were determined by western blot analysis. Both ZIKV E and NS1 protein expression were dramatically reduced by approximately 70–80% by GRP78 siRNA mediated gene silencing as compared to the non-relevant siRNA transfection control (Fig. 9D–F). We note that while the level of infection was reduced by approximately 50% (Fig. 9A), a somewhat greater effect (approximately 66% reduction) was seen in virus titer (Fig. 9B), suggesting that virus production is less efficient in cells with even partial knockdown of GRP78.

The effect of down-regulation of GPR78 to ZIKV binding to the host cell were determined by incubation the siRNA transfected cells with ZIKV-T at m.o.i. of 5 or 200 at 4 °C for 1 h, as this low temperature allows virus attachment onto the cell without the internalization. In addition, the level of viral binding, internalization and entry into host cells was investigated by incubation of siRNA transfected cells with ZIKV at m.o.i. of 5 at 37 °C for 2 h. After these incubations, the unbound virus was removed by washing with ice cold 1 × PBS for 3 times. The level of virus binding or binding, internalization, and entry into the host cells was quantitated by real time PCR. From detected results, there were no significant difference of ZIKV genome copy number when compared between GRP78 siRNA transfected cells and GFP transfected control (Supplemental Figure S1).

### Induction of ER stress, UPR and apoptosis during ZIKV infection

**Figure 10:**
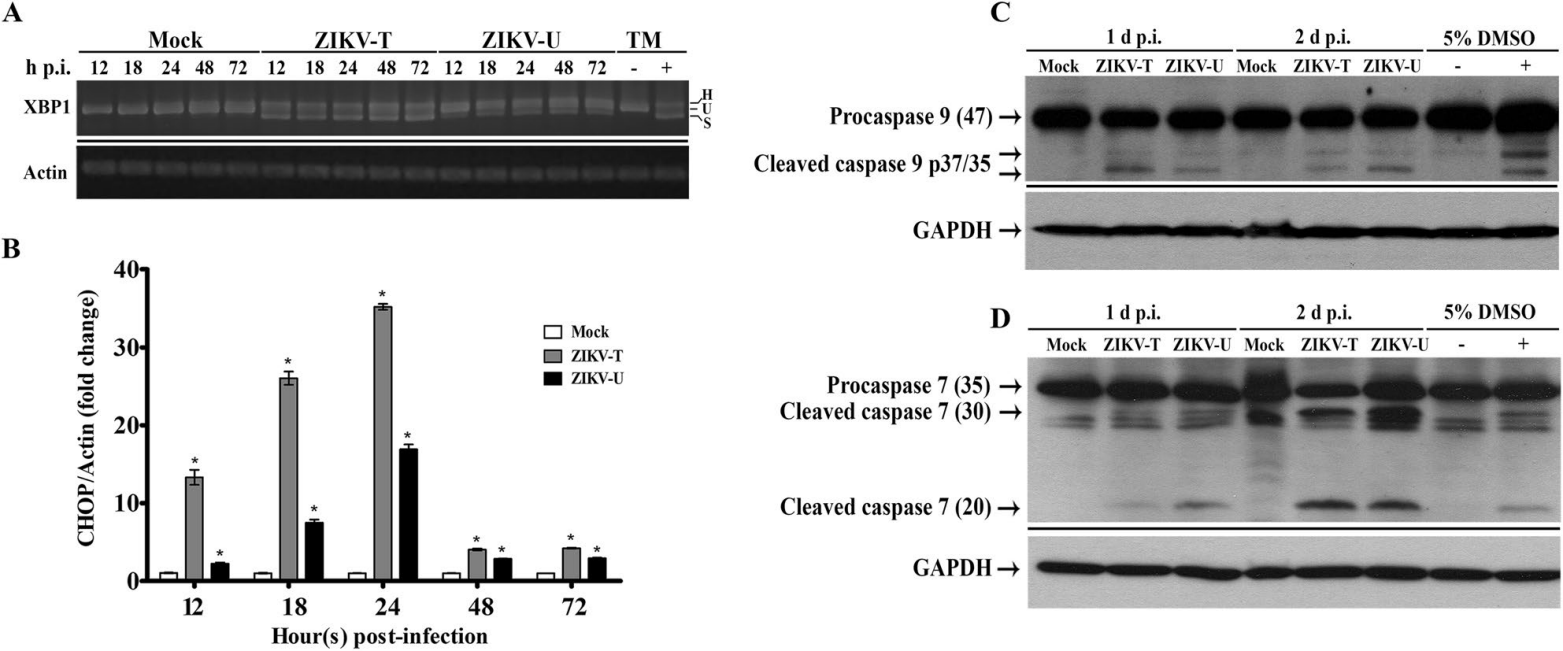
Activation of the UPR and induction of apoptosis during ZIKV infection. A549 cells were mock-infected or infected with ZIKV-T or ZIKV-U at MOI 5 or 2, respectively. At various times as indicated total RNA was extracted and examined for (**A**) the presence of the unspliced (U), spliced (S) and heteroduplex (h) forms of XBP1 mRNA using RT-PCR, with tunicamycin (TM, 4 μg/ml) treated cells as a splicing control and actin as internal control. The PCR products were separated by electrophoresis on 2.0% agarose gels and different PCR experiments are separated by a continuous black line, or (**B**) expression of CHOP mRNA using quantitative real time PCR. The relative expression levels of CHOP mRNA were normalized against actin using the comparative CT method (2^−ΔΔCT^ method). Error bars represent SEM. (*p value < 0.05). Total protein was collected and subjected to western blot analysis to determine the proteolytic processing of (**C**) caspase 9, and (**D**) caspase 7. GAPDH was used as an internal loading control. Cells treated or untreated with tunicamycin or DMSO were included as positive and negative controls. Different probings of the same filter are separated by a continuous black line and uncropped blots are presented in Supplemental materials.

GRP78 is the master regulator of the UPR, and GRP78 mediates the UPR through its interactions with three ER resident proteins, PERK, ATF6 and IRE1^33^. Under non-stress conditions GRP78 binds to PERK, ATF6 and IRE1, while under stress conditions these molecules are released from GRP78 and they subsequently mediate the UPR by down-regulating protein translation, increasing protein degradation and up-regulating the chaperone capacity of the ER^33^. IRE1 is serine/threonine kinase that possesses endonuclease activity. Upon release from GRP78 IRE1 mediates the removal of 26 nucleotides of the X-box binding protein 1 (XBP1) mRNA resulting in a transcription factor that regulates the expression of genes involved in the UPR. To determine if there was activation of the UPR, specific primers were used to examine the XBP1 mRNA to look for IRE1 mediated splicing of XBP1. Results (Fig. 10A) show that the spliced XBP1 message was observed in ZIKV infected cells, while no splicing of XBP1 was seen in control (mock) infections, showing induction of the UPR in response to ZIKV infection.

While the UPR is a pro-survival mechanism that attempts to restore cellular homeostasis^33^, prolonged ER stress is known to result in the induction of apoptosis via a PERK-eukaryotic initiation factor 2 (eIF2)-CCAAT/-enhancer-binding protein homologous protein (CHOP) pathway^52^. To determine whether CHOP was induced by ZIKV infection the level of CHOP mRNA was determined by real time RT-PCR. Results (Fig. 10B) showed that expression of CHOP was significantly increased as a consequence of infection with both ZIKV-T and ZIKV-U as compared with mock infected cells. These results suggest the possibility of ER stress induced cell death in response to ZIKV infection by which CHOP induces apoptosis through the intrinsic apoptosis pathway. To further study the induction of intrinsic apoptosis during ZIKV infection, the activation of caspase 9, which plays a key role in the intrinsic apoptosis pathway and the activation of the execution caspase, caspase 7 were examined by western blot analysis. Results (Fig. 10C,D) showed that the specific cleavage forms of active caspase 7 and caspase 9 were detected in cells infected with ZIKV on 1 day and 2 day post infection, implying the induction of ER stress mediated intrinsic apoptosis in response to ZIKV infection.

## Discussion

ZIKV is a newly emerged mosquito-borne flavivirus whose transmission has recently been documented in Africa, the Pacific Islands, Asia and the Americas^53^. Although most ZIKV infections in humans are asymptomatic or result in mild flu-like symptoms^9^, ZIKV infection can cause serious symptoms including congenital disorders and microcephaly in infants^54^ as well as Guillain–Barré syndrome^55^, myelitis^56^ and meningoencephalitis^57^ in adults. The alarming association of ZIKV infection and neurological impact presents a threat to many populations around the world as there is no licensed vaccine available, nor is there an effective treatment. ZIKV infection remains poorly understood, and a better understanding of the molecular mechanisms of ZIKV host cell interactions is required to identify novel therapeutic targets. The ZIKV E protein is both the major antigenic determinant as well as the protein responsible for receptor binding, and likely plays a role in modulating the host cell machinery to generate an environment favoring viral replication. Thus, this study sought to identify ZIKV E protein interacting proteins using a yeast two-hybrid assay and a human brain cDNA library. Because of cytotoxicity of the full length protein, the yeast-2-hybrid was undertaken using only domain III (Z-EIII), which primarily functions in receptor binding during viral entry and a total of 21 candidate interacting proteins were identified.

Ontological analysis of the identified interacting proteins showed that the identified proteins belonged to diverse pathways and were implicated in a number of biological processes. Proteins of note included DNAJ1B, a molecular chaperone involved in protein folding and protein oligomer assembly. This protein binds with N-terminal ATPase domain of Hsp70 and regulates the ATPase activity of Hsp70 in order to promote protein folding and prevent aggregation of misfolded protein^58^ and the Hsp70 network has been identified as critical in regulating both DENV^59^ and JEV^60^ replication. Interestingly, Hsp70 has recently been shown to co-purify with the ZIKV virion^61^ suggesting a possible interaction between Hsp70 and ZIKV E protein, although such an interaction was not detected in this study. However, as noted the yeast-2-hybrid experiment was undertaken with ZIKV E protein domain III only, and so it is possible that Hsp70 interacts with other domains of ZIKV E protein. Interestingly, another chaperone interacting protein was also identified, as cysteine and histidine rich domain containing 1 (CHORDC1) has been proposed to act as a co-chaperone for Hsp90^62^.

Interestingly, two units of the Na(+)/K(+)-ATPase glycoprotein subunit (subunits beta 1 and beta 3) were identified as interacting proteins. The Na+/K+-ATPase is an integral membrane protein responsible for establishing and maintaining the electrochemical gradients of sodium and potassium ions across the plasma membrane^63^. We are unaware of any reports implicating this protein in ZIKV replication, however one study has shown that inhibition of the Na+/K+-ATPase results in a 50-fold inhibition of DENV production, but not RNA replication^64^. This interaction merits further investigation.

Amongst the Z-EIII interacting proteins identified (Table 1), glucose regulated proteins 78 (GRP78) was identified as particularly worth more details investigation. GRP78 is a multifunctional regulator of endoplasmic reticulum homeostasis which plays critical roles in protein processing, protein quality control, and works as the master control of the UPR^31,65,66^. Moreover, previous studies have reported chaperone protein GRP78 as an interacting protein of the E protein of other flaviviruses, namely DENV^21–24^ and JEV^25,26^. More recently, GRP78 was identified as a protein that interacts with ZIKV E protein in pulldown experiments^67^, a result consistent with our identification of the same interaction in this study. Royle and colleagues also reported effects on ZIKV virus proteins and titers as a consequence of GRP78 depletion^67^. In this study, the interaction between ZIKV E protein and GRP78 was confirmed by both co-immunoprecipitation and reciprocal co-immunoprecipitation in ZIKV infected cells, and the interaction was shown to occur for both ZIKV isolates examined.

Additionally, GRP78 have been proposed to play a role as DENV receptor mediating DENV entry into HepG2 human hepatoma cell^21^ and to serve as a cellular receptor for JEV on neuro-2a neuroblastoma cells, mouse primary neuron cells and Huh7 human hepatoma cells^26^. More recently, GRP78 was defined as a receptor on BHK21 Syrian hamster fibroblast cell for duck Tembusu virus (TMUV, Flavivirus)^68^. In this study, GRP78 was identified as a possible cell surface expressed receptor for ZIKV entry to A549 cell as demonstrated by cell surface co-localization of GRP78 and ZIKV E protein, together with a significant reduction in both infection level and virus production when infection took place in the presence of anti GRP78 N-terminal antibodies. We note that infection was inhibited by pre-incubation with an antibody directed against the N-terminus of GRP78, but not an antibody directed against the C-terminus, a result consistent with our previous study for DENV 2^21^, and would be consistent with the reduction of internalization resulting from inhibition of the ATPase activity required for substrate binding and release^69^. Furthermore, GRP78 knockdown by siRNA-mediated gene knockdown resulted a reduction of ZIKV infection, replication and production in host cells. However, incomplete inhibition of ZIKV infection in cells pre-treated with an anti GRP78 N-terminal antibody and no effect of down regulation of GRP78 to viral binding or/and entry level may suggest that ZIKV uses multiple molecules during entry into the cell, consistent with previous studies that have characterized AXL as ZIKV receptor on A549 cells^17^. Alternatively, it is possible that the downregulation of GRP78 by only around 50% was insufficient to see an effect in cell surface binding. Moreover, given the strong upregulation of GRP78 seen in ZIKV infection, it is likely that the downregulation is somewhat transient.

Many reports have shown that activation of the UPR is a common consequence of flavivirus infection, likely via the influx of viral protein into ER^70,71^ and the induction of GRP78 during ER stress is considered a hallmark of the UPR^72^. The UPR is regulated through the binding and release of GRP78 from UPR signaling proteins including PERK, ATF6 and IRE-1^73^. Our study showed the intracellular co-localization of GRP78 and ZIKV E protein, as well as an increase in GRP78 at both the transcriptional and translational levels in ZIKV infected cells. Thus the activation of a UPR sensor namely, IRE1 was investigated. The results showed that ZIKV infection induced IRE1 mediated XBP1 splicing. It is believed that several flaviviruses take advantage from the activation of IRE1-XBP1 branch of the UPR to relieve cell cytotoxicity^74^. Interestingly, a previous study has demonstrated that ZIKV induced eIF2a phosphorylation via activated PERK^75^, indicating activation of multiple branches of the UPR. In particular, activation of the IRE1 pathway has been previously reported in ZIKV infection^76,77^, consistent with the results reported here. Under prolonged ER stress, cell apoptosis is induced through the activity of the pro-apoptotic transcription factor, CHOP which is a downstream gene in the PERK signaling pathway^52^. A significant up-regulation of CHOP mRNA was observed with infection by both strains of ZIKV. In addition, we have shown that CHOP induced cell apoptosis through activation of the intrinsic apoptosis pathway in response to ZIKV infection as evidenced by activation of caspase 9. Previous reports have shown that DENV infection in GRP78-inhibited cells resulted in down-regulation of CHOP and attenuation of ER stress resulting in decreased viral replication^78^. Similarly, another study demonstrated that ER stress activation was associated with neurogenesis deficits, and suggested that relieving ER stress activation may lead to prevention of ZIKV-related microcephaly^79^.

Overall, this study has identified a number of ZIKV E proteins interacting proteins, many of which have the potential for further investigation. In particular, we have demonstrated the interaction of ZIKV E protein with GRP78 and shown that this is a functional interaction that can facilitate internalization of ZIKV into cells, and is required for effective ZIKV replication. GRP78 is emerging as a critical protein in flavivirus replication, and as a potential therapeutic target.

## Supplementary Information

Supplementary Information.

## Data Availability

All data generated or analysed during this study are included in this published article (and its Supplementary Information files).
