# Supplementary material for: A functional interaction between GRP78 and Zika virus E protein
Source: Sci Rep. 2021 Jan 11;11:393. doi: 10.1038/s41598-020-79803-z (PMC7801745; doi:10.1038/s41598-020-79803-z)

Supplemental materials

Supplemental Tables and uncropped blots

A functional interaction between GRP78 and Zika virus E protein

Sarawut Khongwichit[1†], Wannapa Sornjai[1†], Kunlakanya Jitobaom[1], Mingkwan Greenwood[2], Michael P. Greenwood[2], Atitaya Hitakarun[1], Nitwara Wikan[1], David Murphy[2] and Duncan R. Smith[1]*

[1] Institute of Molecular Biosciences, Mahidol University Thailand

[2]Bristol Medical School: Translational Health Sciences, Dorothy Hodgkin Building, University of Bristol, Bristol, United Kingdom

[†]These authors contributed equally.

* Corresponding author. Postal address: Molecular Pathology Laboratory, Institute of Molecular Biosciences, Mahidol University, 25/25 Phuttamonthon 4 Road, Salaya, Nakhon Pathom, 73170, Thailand. Phone: 66 (0) 2441-9003 to 7. Fax: 66 (0) 2441-1013. E-mail: duncan_r_smith@hotmail.com

Supplemental Table S1. Experimental control for yeast mating with full length ZIKV E protein.

Supplemental Table S2. Functional enrichments in biological processes identified by the STRING bioinformatic analysis software.

Supplemental Figure S1. Effect of GRP78 knockdown to ZIKV binding and entry

Uncropped blots

Supplemental Table S1. Experimental control for yeast mating with full length ZIKV E protein.

	**No. of colony**	**Viability (cfu/ml)**	**No. of screened clones**
**Bait** (SD/-Trp)	1904 (1;10000)	5×10^7	
**Prey** (SD/-Lue)	181(1;10000)	5×10^7	
**Diploid** (SD/-Trp/-Lue)	295 (1;1000)	1.4×10^6	1.4×10^7 clones
**Mating efficiency**	6.7%		

Supplemental Table S2. Functional enrichments in biological processes identified by the STRING bioinformatic analysis software.

Biological Process (GO)			
pathway ID	*pathway description*	*Count**	*FDR*
GO:0032781	Positive regulation of ATPase activity	3	0.00922
GO:1902307	Positive regulation of sodium ion transmembrane transport	3	0.00922
GO:1903278	Positive regulation of sodium ion export from cell	2	0.00922
GO:1903288	Positive regulation of potassium ion import	2	0.00922
GO:0072659	Protein localization to plasma membrane	4	0.0111
GO:1901016	Regulation of potassium ion transmembrane transporter activity	3	0.0111
GO:0036376	Sodium ion export from cell	2	0.0193
GO:0030007	Cellular potassium ion homeostasis	2	0.0252
GO:0032414	Positive regulation of ion transmembrane transporter activity	3	0.0252
GO:0090002	Establishment of protein localization to plasma membrane	3	0.0303
GO:1903894	Regulation of IRE1-mediated unfolded protein response	2	0.0303
GO:0006883	Cellular sodium ion homeostasis	2	0.0324
GO:0032507	Maintenance of protein location in cell	3	0.0324
GO:2000651	Positive regulation of sodium ion transmembrane transporter activity	2	0.037
GO:0086009	Membrane repolarization	2	0.0403
GO:1901018	Positive regulation of potassium ion transmembrane transporter activity	2	0.0437

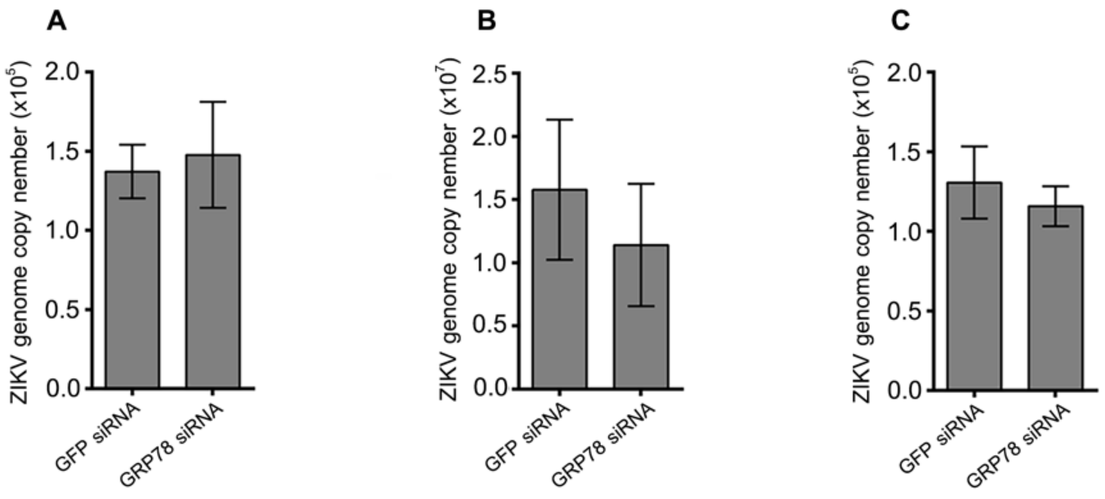

Supplemental Figure S1. Effect of GRP78 knockdown to ZIKV binding and entry

A549 cells were either transfected with GFP siRNA or GRP78 siRNA for 24 h. The transfected cells were incubated with ZIKV at m.o.i. of (A) 5 or (B) 200 at 4°C for 1 h. (C) Transfected cells were incubated with ZIKV at m.o.i. of 5 at 37°C with 5% CO_2 for 2 h. Unbound virus was removed from cells and then ZIKV genome copy number were quantitated by real-time PCR. The graphs are representative of three independent biological replicates. Error bars represent SD.

Figure 3B GRP78 protein expression

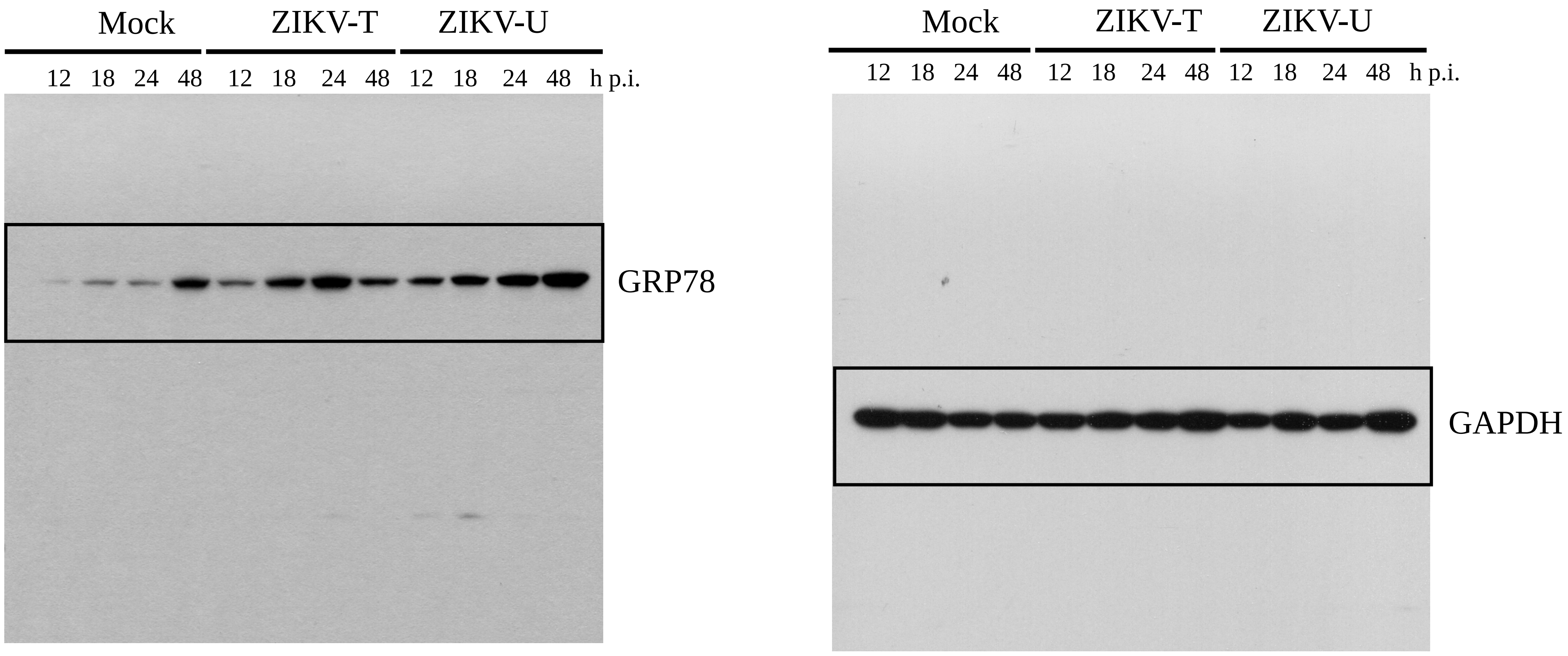

Figure 3B GRP78 protein expression

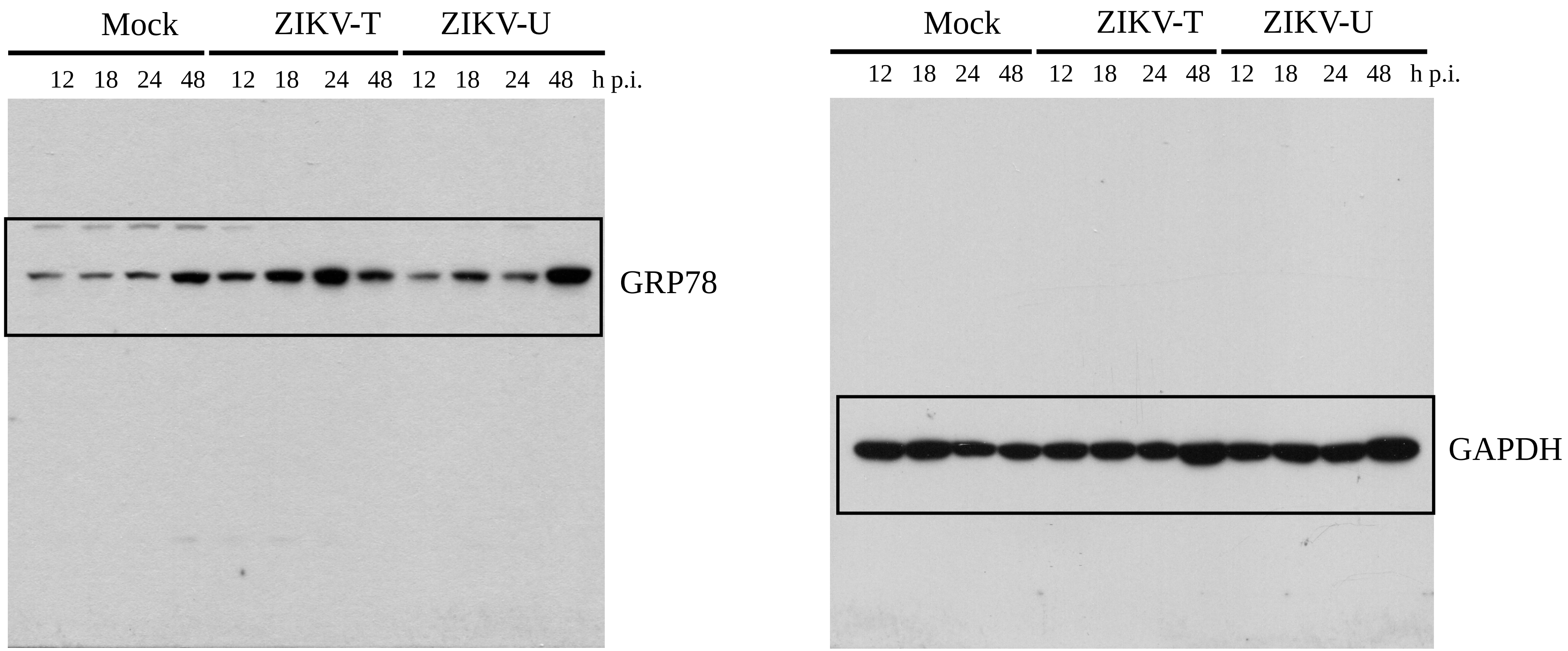

Figure 3B GRP78 protein expression

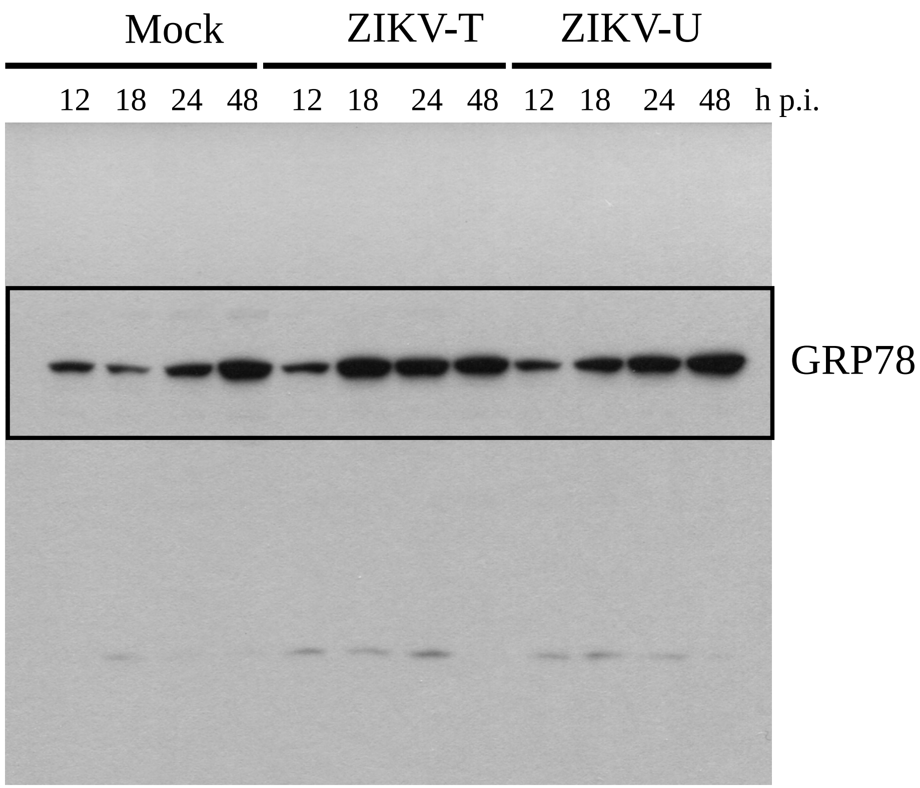

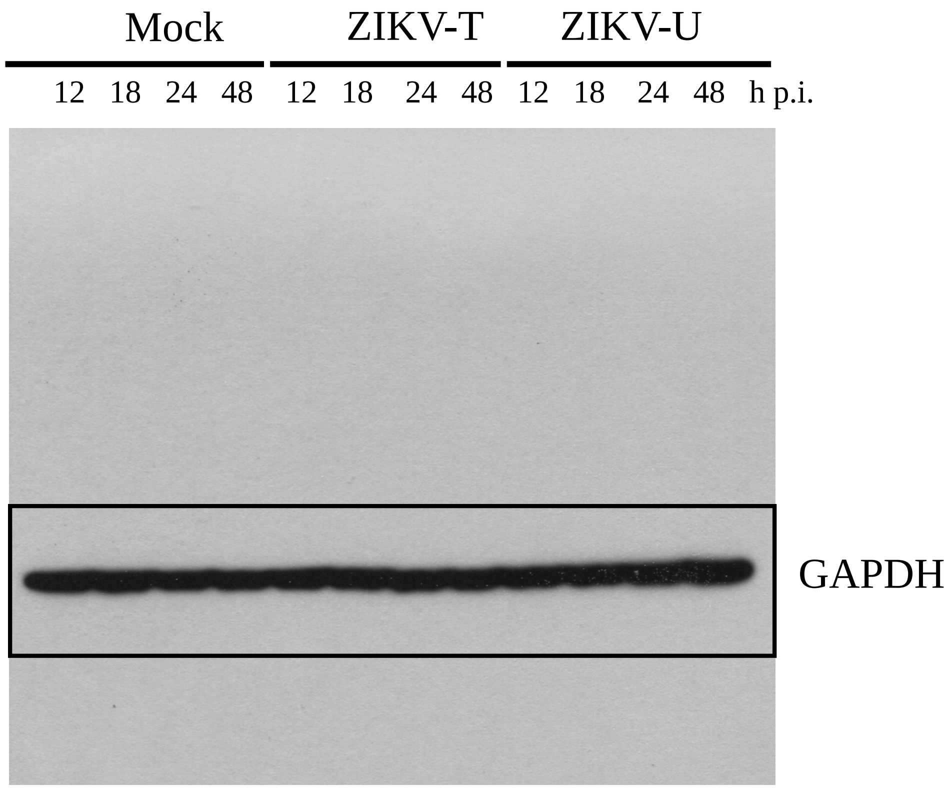

Figure 4A Co-immunoprecipitation assay

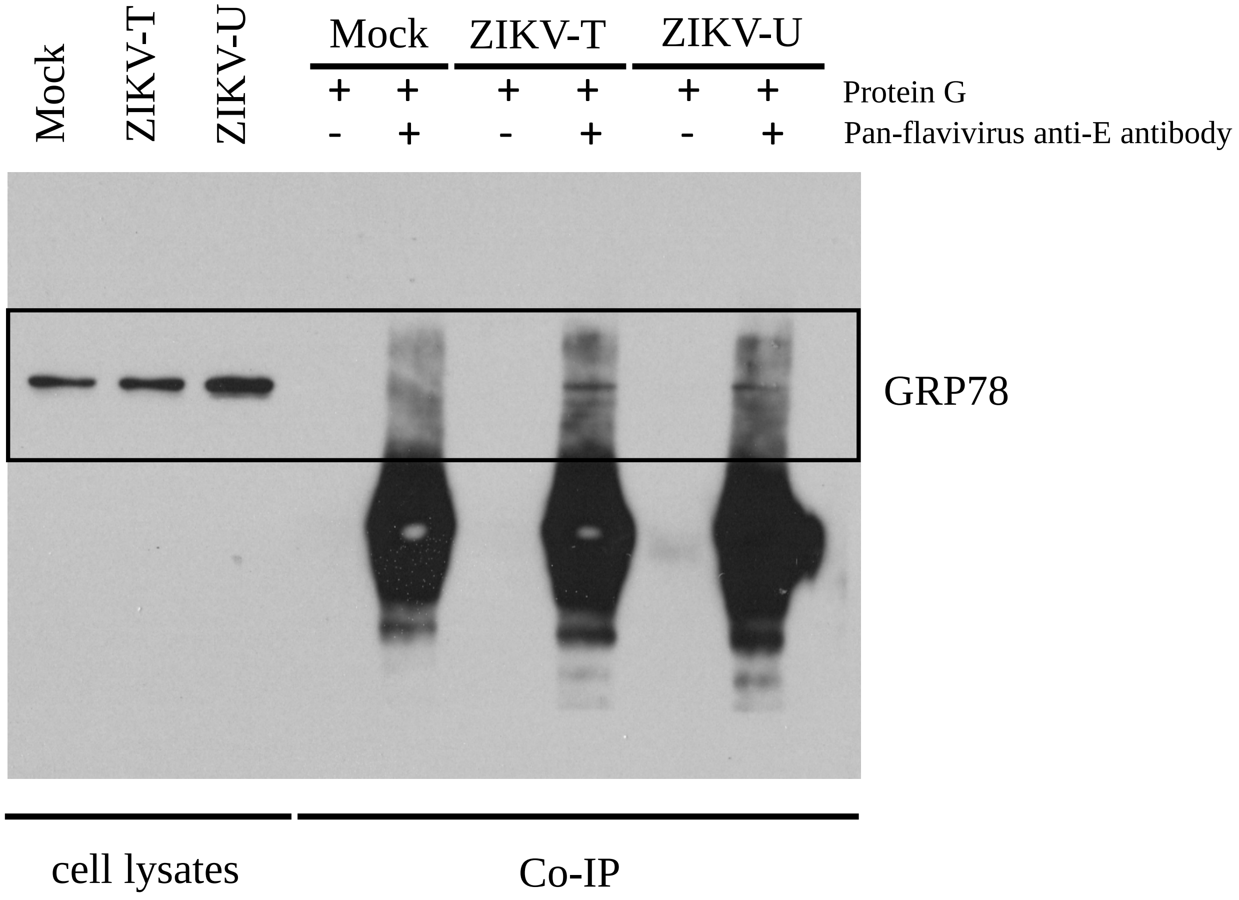

Figure 4A Immunoprecipitation assay

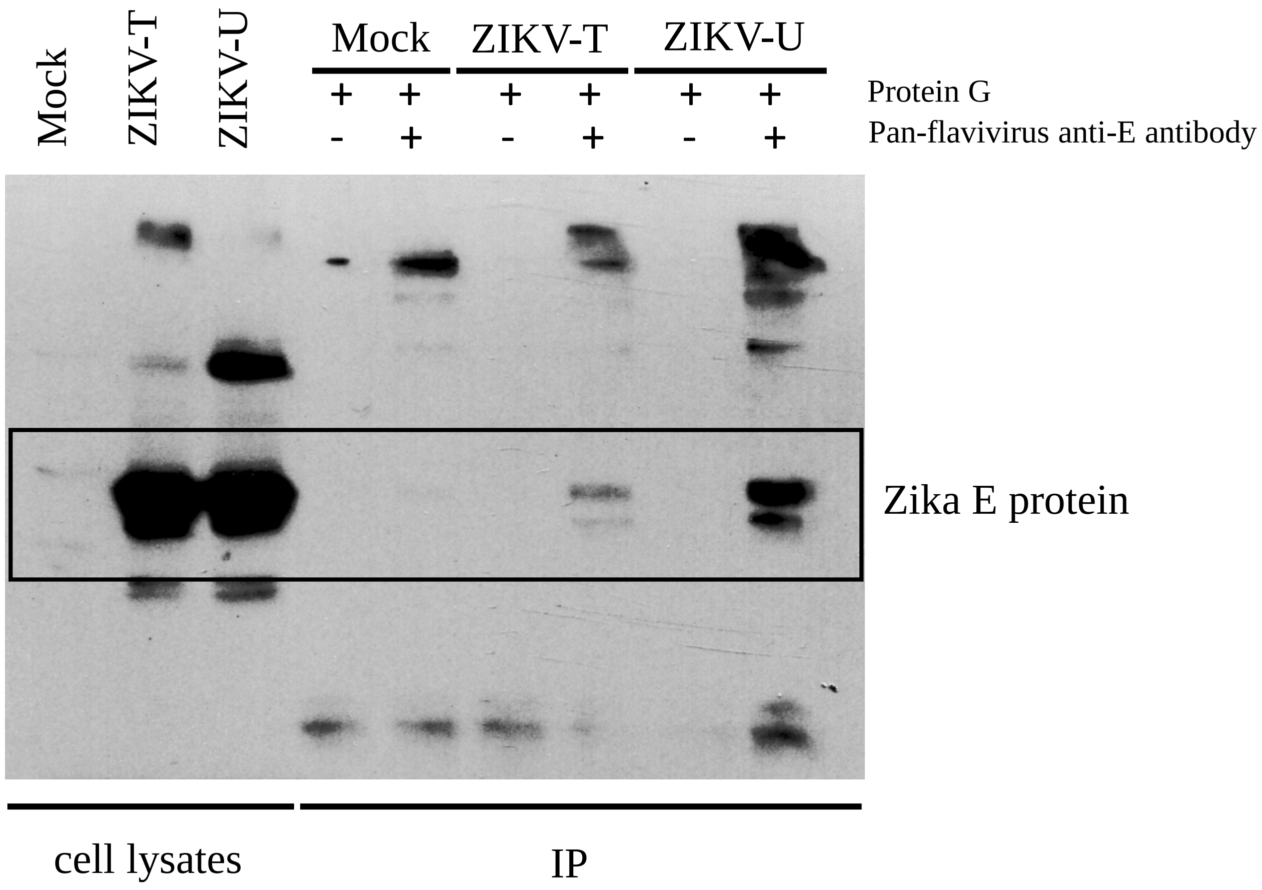

Figure 4B Reverse co-immunoprecipitation

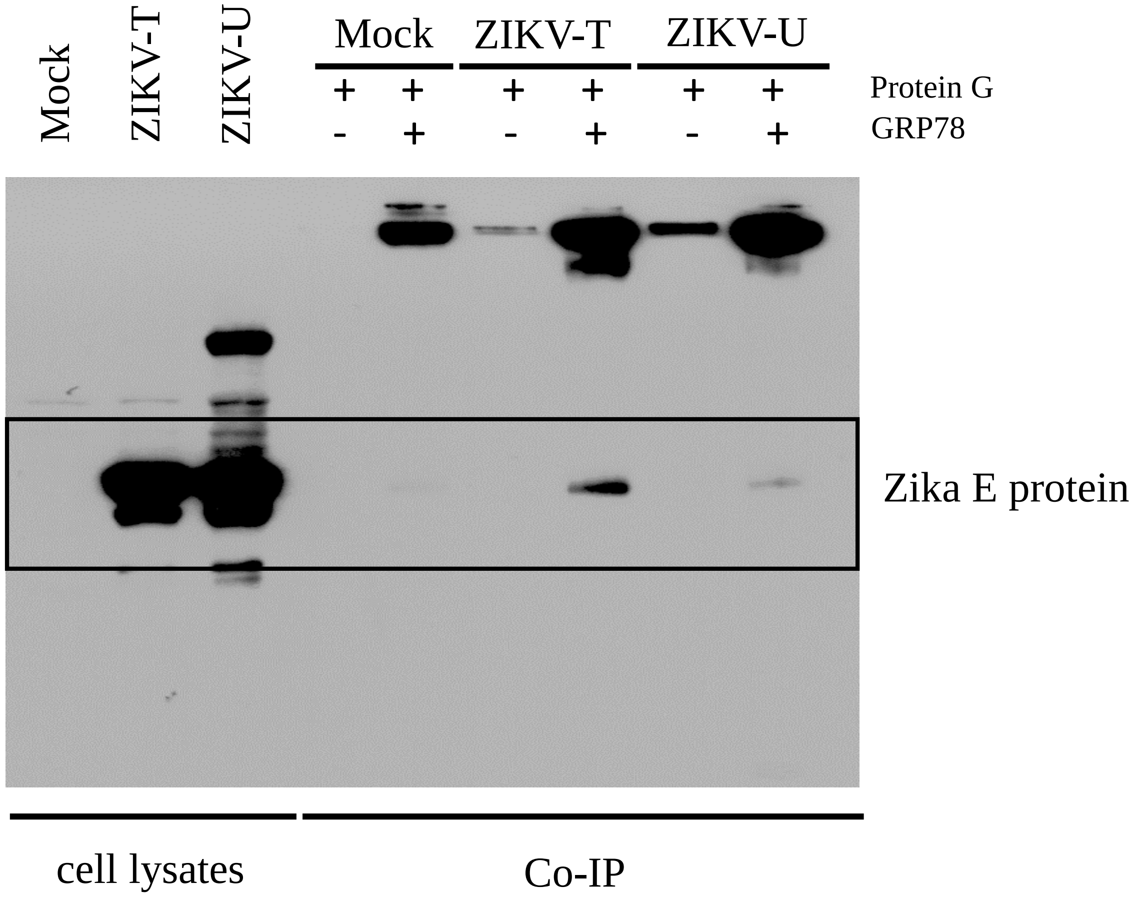

Figure 4B Immunoprecipitation assay

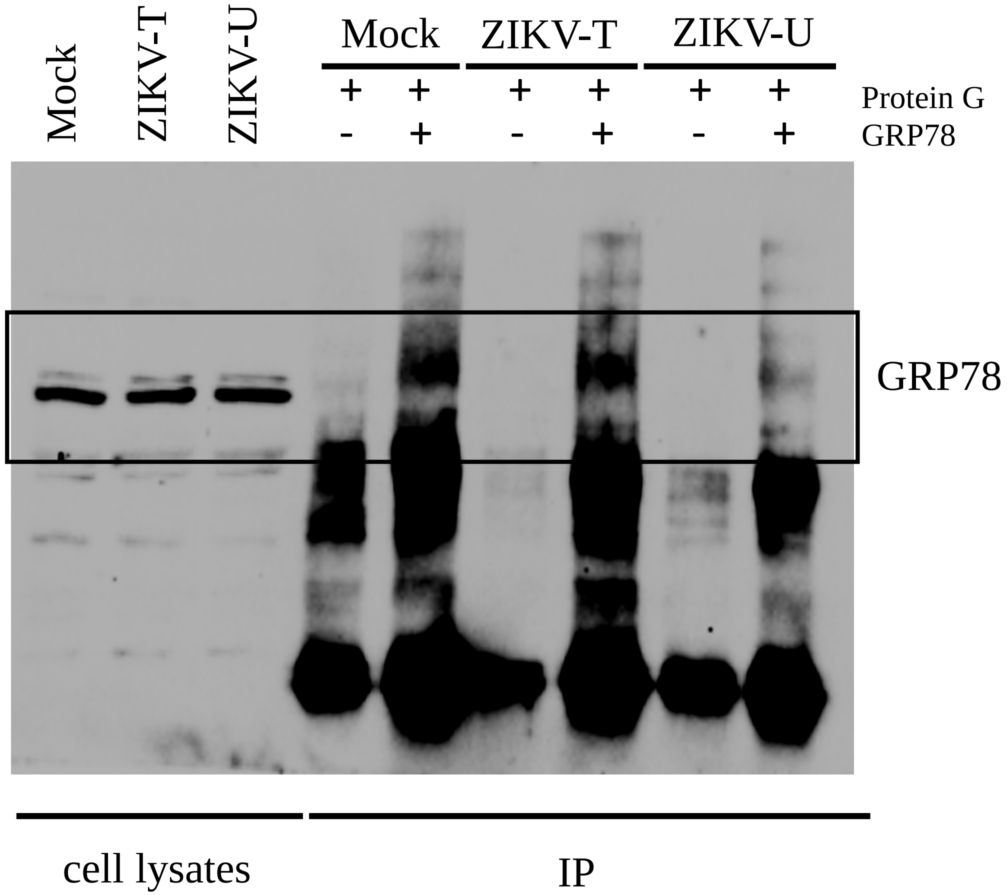

Figure 8B GRP78 protein at 24 h post siRNA transfection

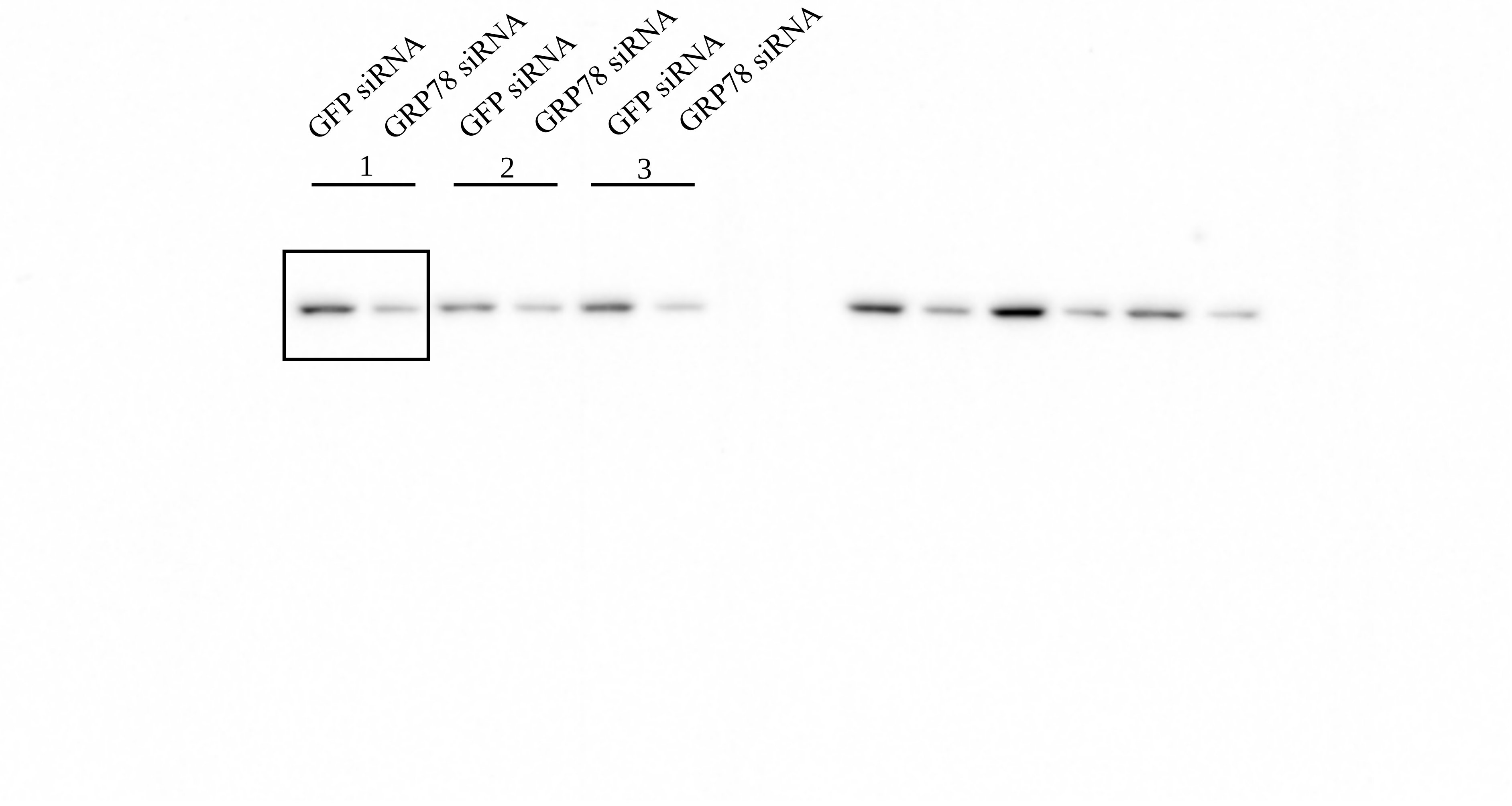

Figure 8B β-actin protein at 24 h post siRNA transfection

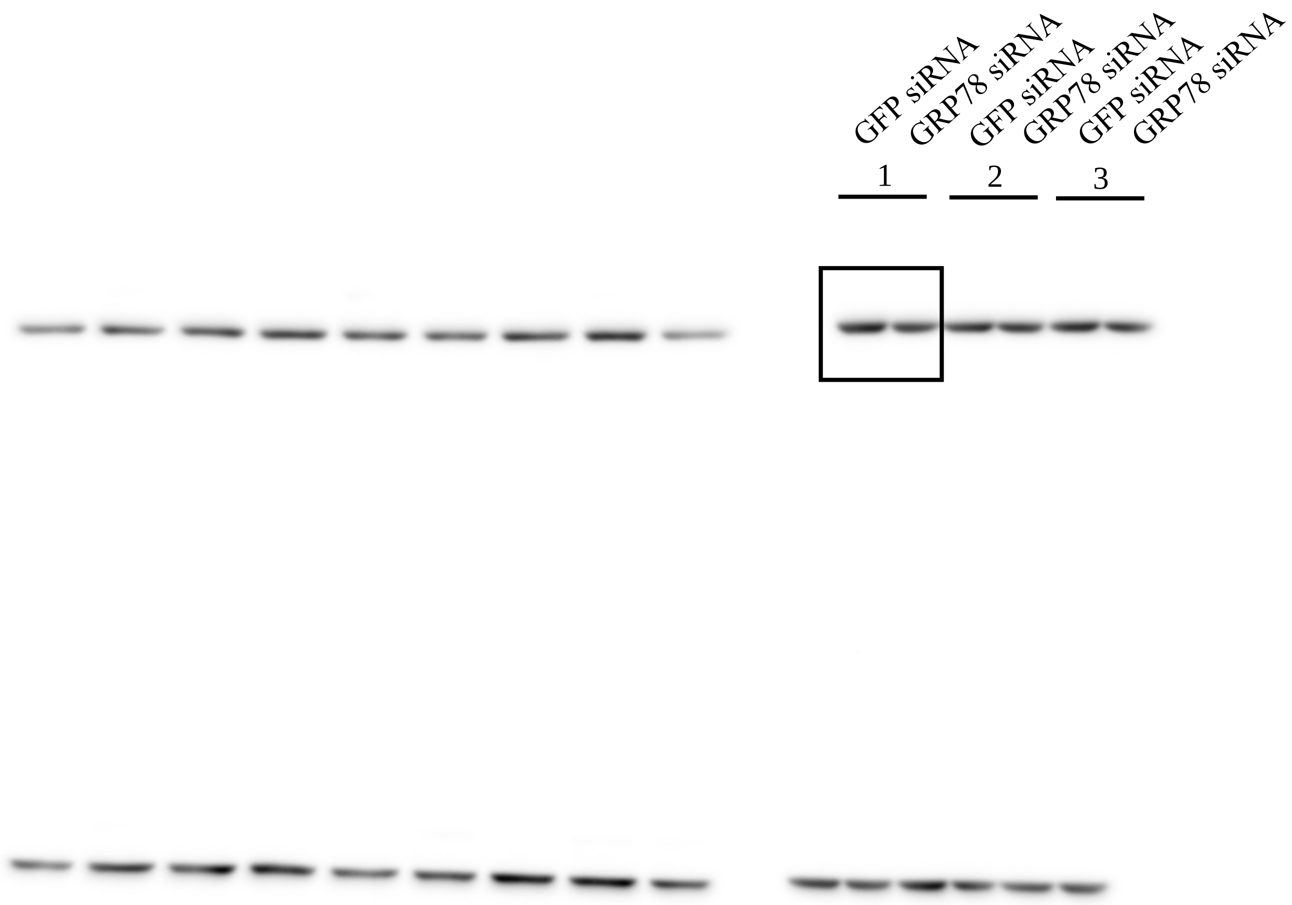

Figure 9D ZIKV E protein at 24 h post infection of siRNA transfected cells

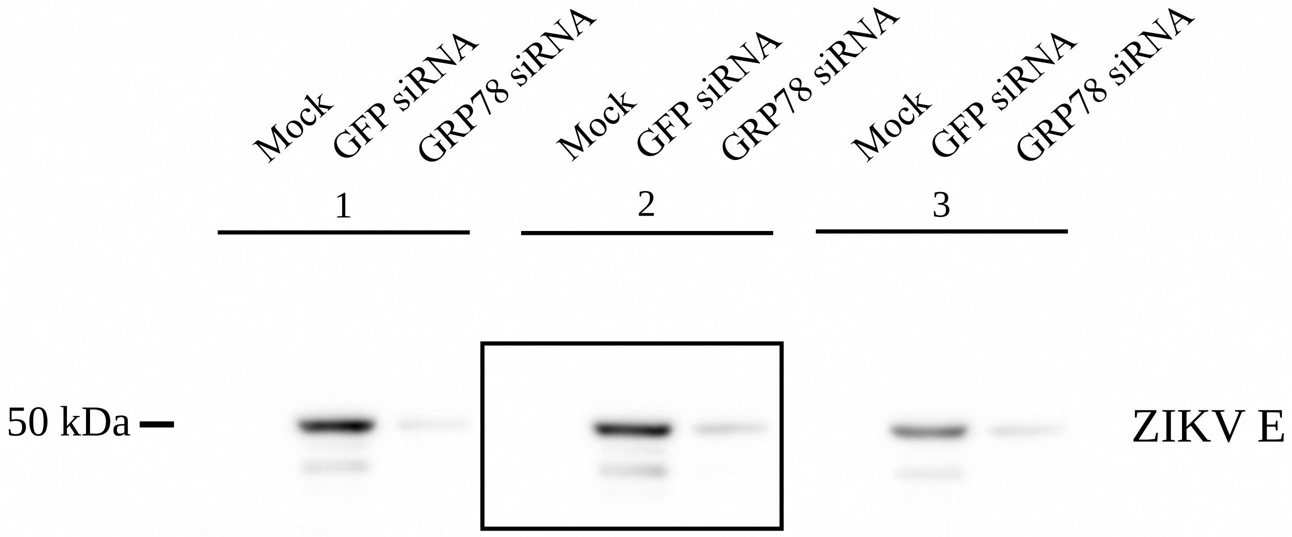

Figure 9D ZIKV NS1 protein at 24 h post infection of siRNA transfected cells

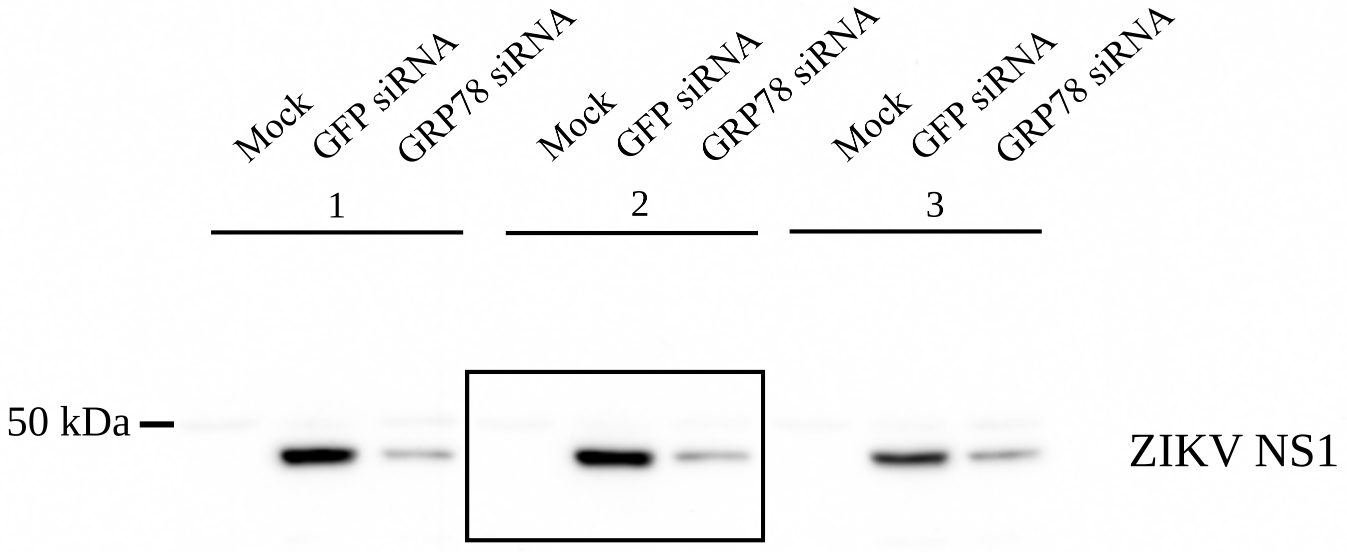

Figure 9D β-actin protein at 24 h post infection of siRNA transfected cells

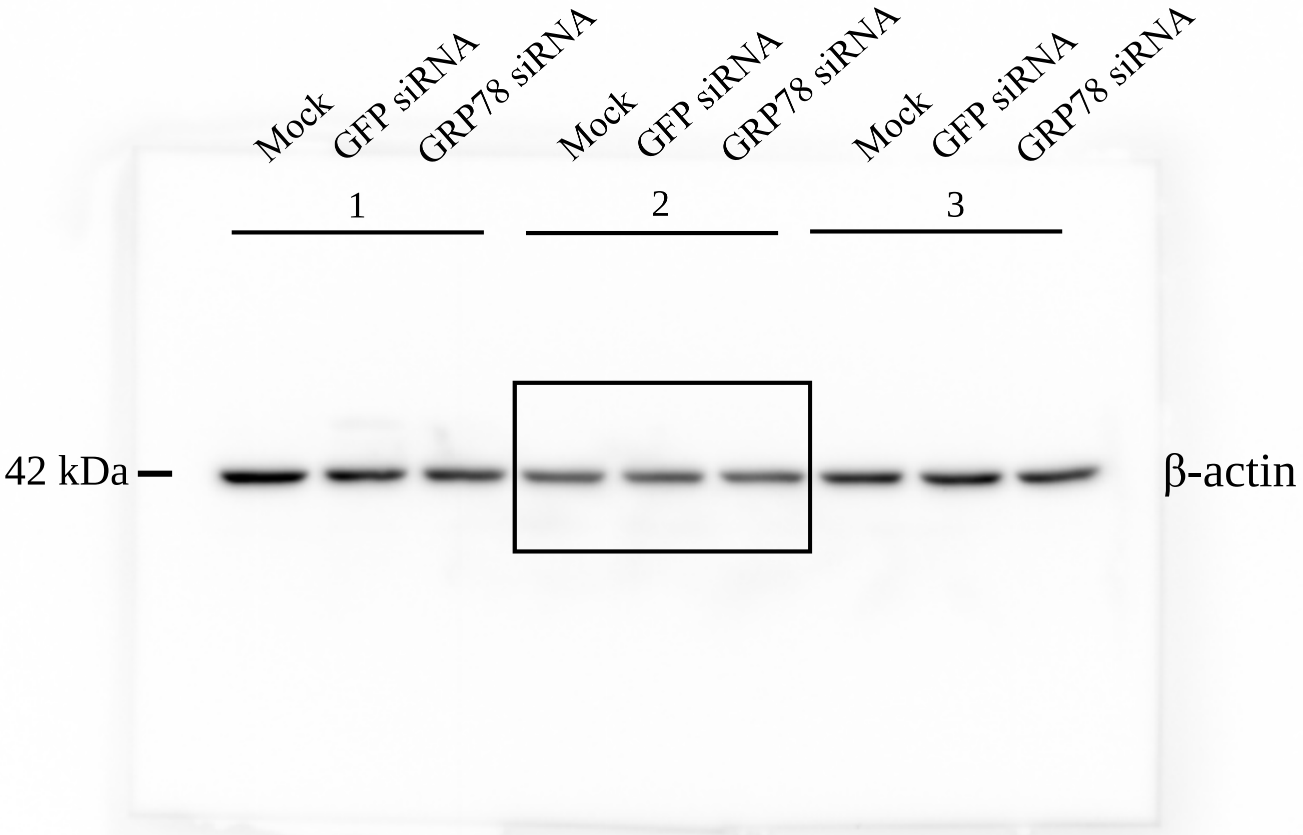

Figure 10C Activation of caspase 9 in ZIKV infection in A549 cells

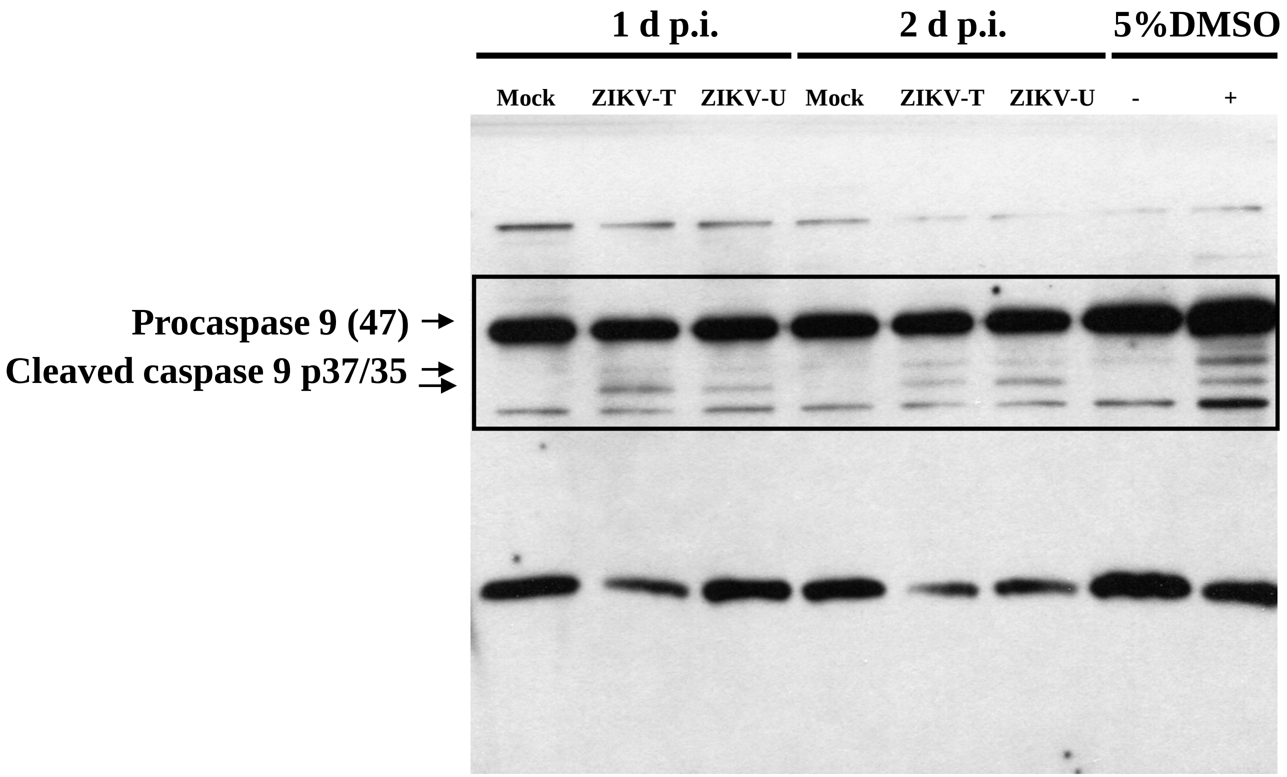

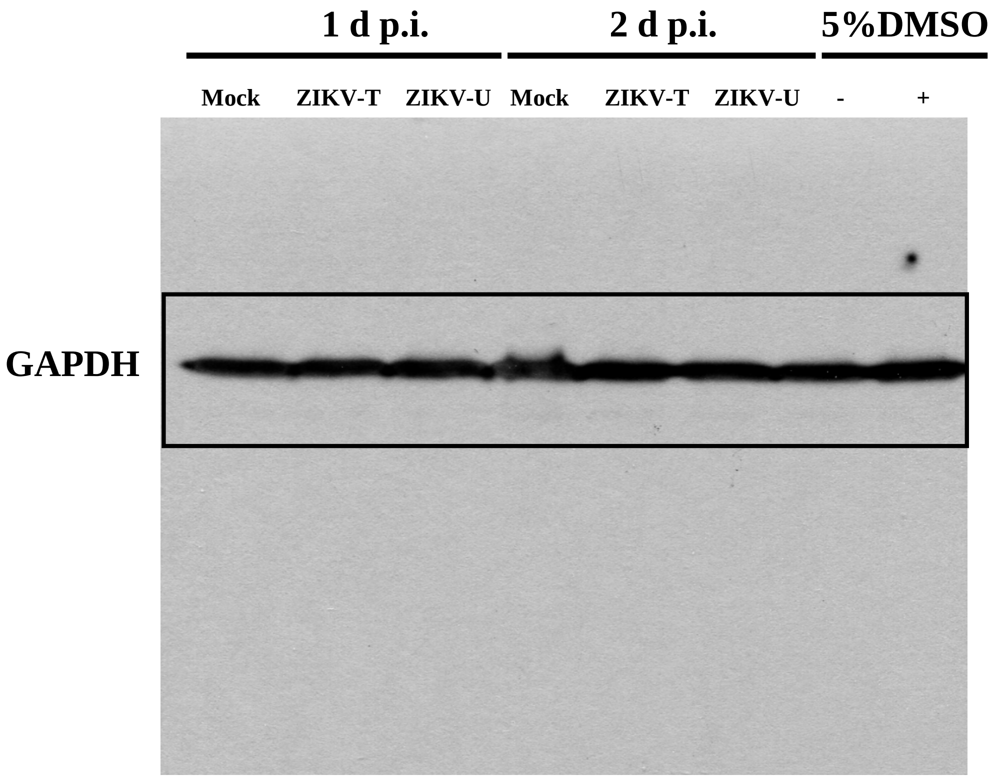

Figure 10D Activation of caspase 7 in ZIKV infection in A549 cells

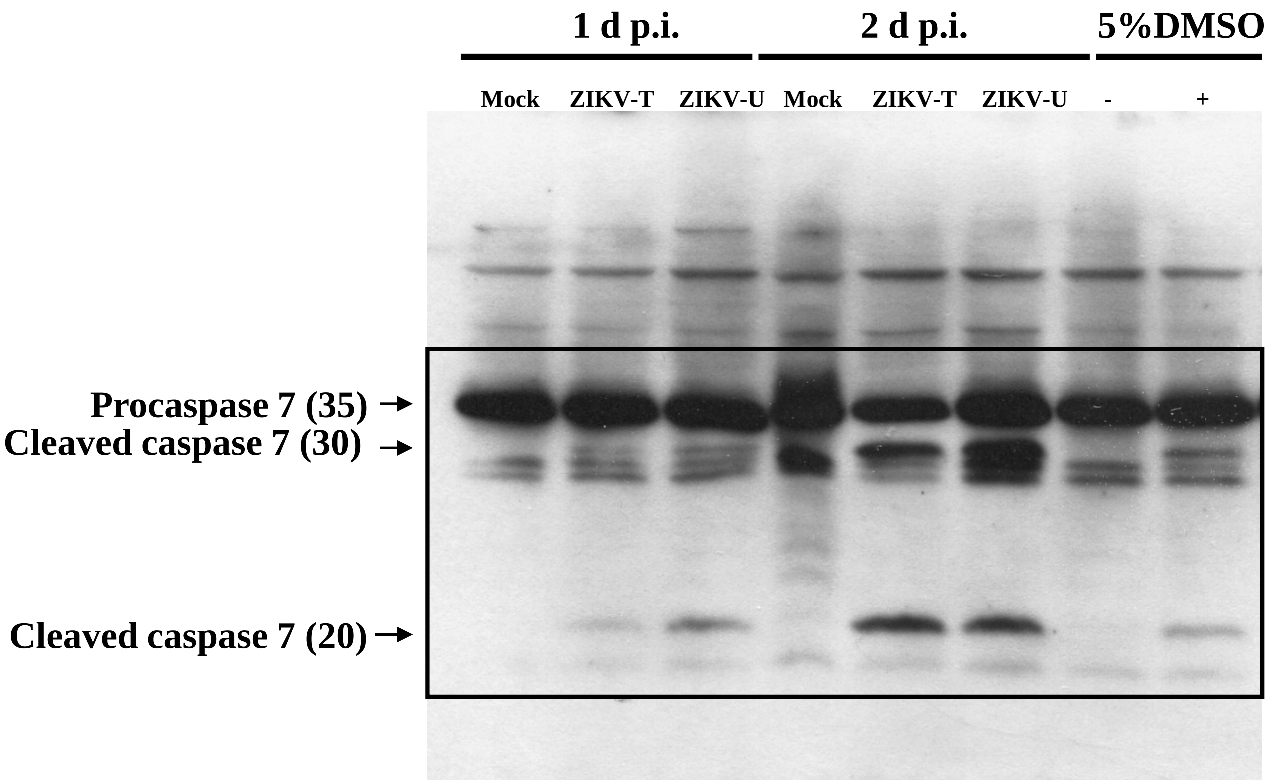

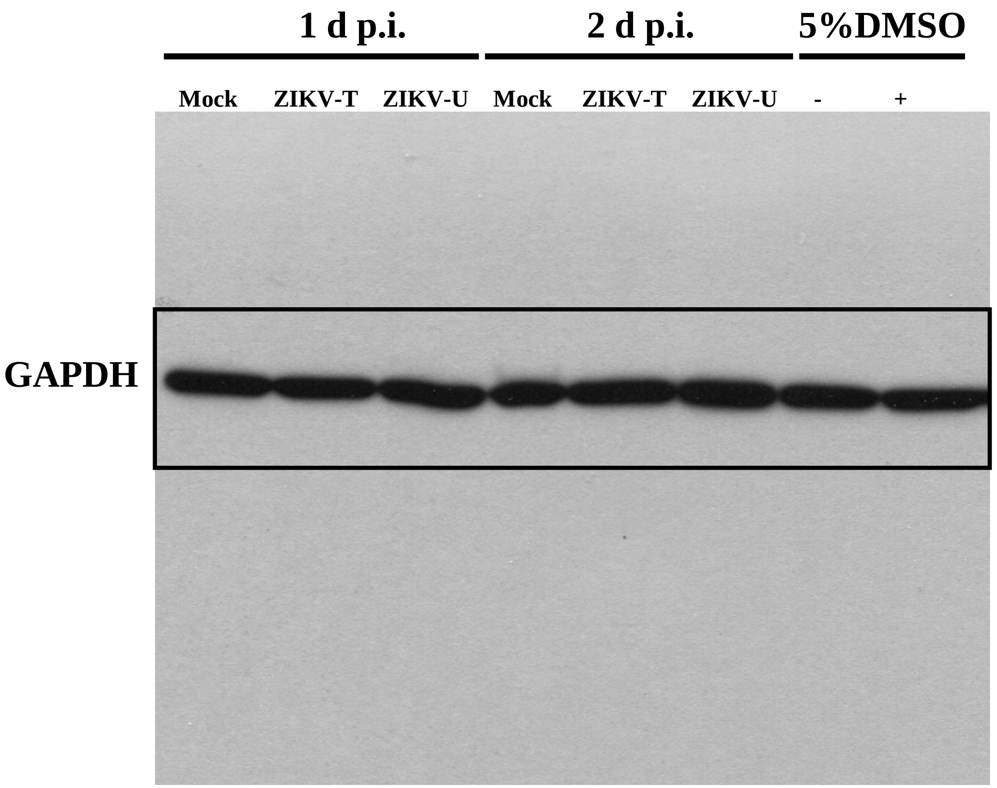

Supplement: Supplementary file 1 — Supplementary Information. [file 41598_2020_79803_MOESM1_ESM.pdf]
